# Macrocyclization: Enhancing Drug-like Properties of Discoidin Domain Receptor Kinase Inhibitors

**DOI:** 10.1021/acsmedchemlett.4c00611

**Published:** 2025-04-07

**Authors:** Laura Carzaniga, Roberta Mazzucato, Valentina Mileo, Andrea Rizzi, Maura Vallaro, Giuseppe Ermondi, Silvia Cattani, Andrea Secchi, Giulia Caron

**Affiliations:** # 18869 Chiesi Farmaceutici, Corporate Preclinical R&D, Research Center, Largo Belloli 11/A, 43122 Parma, Italy; & 9314 University of Torino, Molecular Biotechnology and Health Sciences Dept., CASS MedChem, Piazza Nizza 44bis, 10126 Torino, Italy; @ University of Parma, Department of Chemistry, Life Sciences and Environmental Sustainability, Parco Area delle Scienze 17/A, 43123 Parma, Italy

**Keywords:** discoidin domain receptor, fibrosis, chameleonicity, kinase inhibitor macrocycles, experimental physicochemical descriptors, permeability, solubility

## Abstract

Macrocyclization, a well-established strategy for developing ligands against challenging drug targets, was employed to design macrocyclic alternatives to a linear discoidin domain receptor (DDR) inhibitor (**1**) with potential applications in treating fibrotic diseases. This study aimed to enhance the drug-like profile of **1** through innovative design strategies encompassing molecular docking and chameleonicity considerations. These efforts resulted in the synthesis of matched pairs of macrocycles differing in flexibility and linker features. Compound **5a** emerged as a promising lead, exhibiting nanomolar-range activity, significantly improved solubility, and excellent permeability. Comprehensive experimental physicochemical characterization further highlighted the modest impact of ionization, the major role played by lipophilicity (but not polarity) in driving permeability of the investigated matched pairs, and the limitations of traditional 2D computational descriptors in predicting macrocycle ADME-related properties.

## Graphical abstract

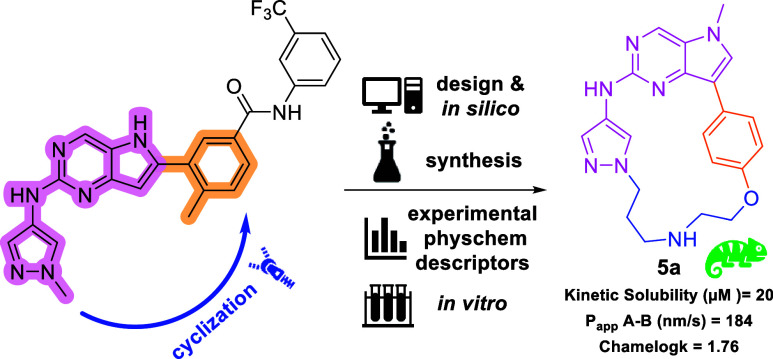

Fibrosis is a pathological condition characterized by the excessive accumulation of extracellular matrix (ECM) components, leading to tissue scarring and organ dysfunction. It is a common feature of various chronic diseases, including liver cirrhosis, pulmonary fibrosis, and systemic sclerosis.^1^ Despite significant advances in understanding the molecular mechanisms underlying fibrosis, effective therapeutic strategies remain limited.

Discoidin domain receptors (DDRs), a unique class of receptor tyrosine kinases, have emerged as critical regulators of ECM remodeling and fibrosis.^2^ Recent studies have highlighted the pivotal role of DDRs in the pathogenesis of fibrotic diseases by enhancing the activation and proliferation of myofibroblasts^3^ and through dysregulation of fibroblast function and ECM production.^4^ The aberrant activation of DDRs in fibrotic tissues suggests that these receptors may serve as potential therapeutic targets.

Macrocycles (MCs), compounds characterized by the presence of a ring containing a minimum of 12 heavy atoms,^5^ represent a still-underexploited chemical modality in drug discovery. Their semirigid, prearranged framework makes MCs well-suited for tackling challenging drug targets with large, flat, and shallow binding sites.^6–8^ Moreover, the conformational constraints allow MCs to mitigate the internal entropy penalty associated with the transition from an unbound state to a bound state.^6,9^ Additionally, cyclization can enhance physicochemical and absorption, distribution, metabolism, and excretion (ADME) properties, such as solubility, permeability, metabolic stability, and oral bioavailability.^10^

Notwithstanding, MCs’ complex structures introduce hurdles in the overall pharmaceutical development process.^11,12^ Furthermore, optimizing MCs is challenging due to the limited data on how individual structural changes affect physicochemical and ADME profiles.^6^ The impact of structural modifications on their behavior may also be hampered by chameleonicity,^13^ i.e., the capacity of compounds to adapt to the environment through conformational modifications. Chameleonicity is a particularly useful characteristic in macrocyclic space, able to improve the molecular capacity to balance aqueous solubility and passive cell permeability.^6^

Several kinase inhibitor macrocycles (KIMCs), cyclic variants of previously known uncyclized inhibitors, have been developed so far (Figure S1),^14,15^ leveraging the opportunity to identify new, patentable compounds in the crowded intellectual property space of kinase inhibitors. Therefore, cyclization offers the additional advantage of expanding the scope for innovation.^16^

A recent internal program led to the discovery of a novel class of *in vitro* potent linear DDRs inhibitors characterized by a pyridopyrimidine core. They had suboptimal ADME profiles, mostly due to their extremely low solubility, as highlighted by compound **1** (chemical structure in Figure 1 and data in Table 1), which showed a nanomolar activity in the selected cellular system, suggesting that it is capable of crossing cell membranes. However, when permeability was measured in the MDCK model, the obtained value was unreliable due to low recovery, likely caused by poor solubility affecting the assay’s performance (Table 1).

**Figure 1:**
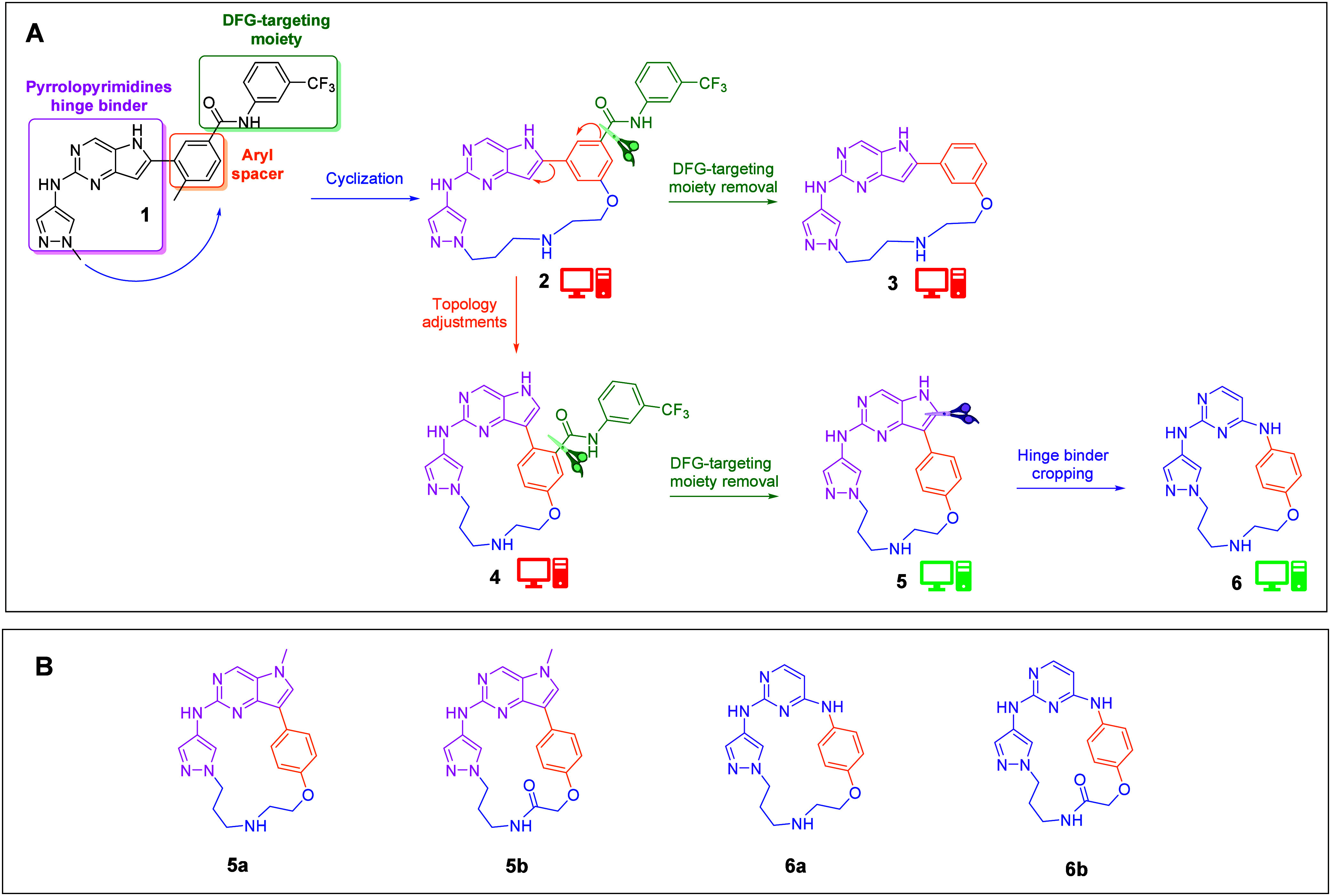
A) KIMCs design strategy and results of docking assessment (red computing unit = low docking score; green computing unit = high docking score). B) Chemical structures of the synthesized cyclized compounds bearing the pyrrolopyrimidine core (**5a** and **5b**) or the amino-pyrimidine core (**6a** and **6b**).

**Table 1:** DDR1 Cellular Activity, Kinetic Solubility, MDCK Permeability, Caco-2 Permeability, and Clearance in Human Microsomes

				Caco-2 P_app_ AB/BA (nm/s)			
Compd	Cell DDR1 IC_50_ (μM)	KS (μM)	WT-MDCK P_app_ AB (nm/s)	with Pgp inhibitor	without Pgp inhibitor	Pgp substrate	CL_int_ h mic[Table-fn t1fn3] (μL/min/mg)
**1**	0.003	<5	ND	ND	ND	ND	19 ± 2.1
**5a**	0.125	20 ± 3	184 ± 6	199 ± 27/135 ± 32	102 ± 15/242 ± 72	NO	80.5 ± 2.7
**5b**	2	16 ± 1	210 ± 8	228 ± 28/159 ± 9	30 ± 6/215 ± 29	YES	88.5 ± 9.6
**6a**	1.25	188 ± 18	58 ± 11	87 ± 19/70 ± 9	19 ± 5/220 ± 96	YES	22.1 ± 1.4
**6b**	>3.15	198 ± 3	48 ± 10	55 ± 2/203 ± 7	6 ± 1/223 ± 29	YES	12.1 ± 0.1

a Clearance in human microsomes (μL/min/mg); ND = not determined due to low mass balance. Thresholds: KS (Kinetic Solubility): high, >150 μM; medium, 50–150 μM; low, <50 μM; MDCK P_app_: high, P_app_ > 100 nm/s; medium, 30 < P_app_ >100 nm/s; low, <30 nm/s; Caco-2 permeability: high, >80 nm/s; medium, 20–80 nm/s; low, <20 nm/s; CL_int_: high, >60 μL/min/mg; medium, 10 < CL_int_ > 60 μL/min/mg; low, <10 μL/min/mg.

Compound **1** was considered a representative example of its chemotype and thus was selected as the prototypical starting point for the exploration of cyclized derivatives. The main aim of this exercise was to obtain macrocyclic alternatives with superior drug-like profiles compared to **1**.

To reach our goal, we first set up the KIMC design strategy schematized in Figure 1. Docking was performed to assess the binding mode and provide guidance on compound prioritization for synthesis. Next, we calculated 2D descriptors to evaluate the predicted molecular properties profile of the selected cyclic derivatives. The synthesized compounds were submitted to *in vitro* characterization, and we also assessed their physicochemical profile with a pool of sophisticated chromatographic descriptors.

We analyzed matched-pair molecules to better understand how structural modifications influence the physicochemical properties and ADME profiles of the MC series. The results are expected to provide preliminary but solid guidelines to be applied to lead optimization in future MC drug discovery programs.

To start, we planned a cyclization (blue arrow Figure 1A) connecting the aryl spacer (orange) with the pyrazolyl group (pink). Leveraging the highly conserved nature of the ATP binding pocket in kinases, our strategy was guided by the structural features of the most advanced compounds (Figure S1), and we designed our molecules in a cyclized conformation featuring a 7-atom linker, resulting in a 19-atom ring size. This specific linker length was determined to be optimal for enhancing binding affinity compared to both longer and shorter analogues (see Supporting Information (SI)). A phenolic handle was selected to connect the cyclizing linker to the aryl core and the methyl in position 4 was removed, facilitating synthetic accessibility (**2**). Next, we removed the DFG-targeting moiety (**3**). Docking assessment revealed low scores for compounds **2** and **3**. Therefore, a topology adjustment was proposed, to minimize clashes and achieve a proper binding mode. To do that, we adjusted the exit vectors of the aryl spacer, transforming the molecular topology into a C-shape that could better accommodate the macrocyclic constraint (**4**). Unfortunately, docking studies showed that even after topology adjustment, the DFG group still hindered interaction with the kinase cavity. Again, we removed it (**5**). Finally, we excised the pyrrolyl ring and repositioned the nitrogen atom to an adjacent location to serve as a connecting handle, thereby creating an amino-pyrimidine core (**6**). This modification aimed to introduce greater flexibility and reduce lipophilicity compared to compounds **5**. Both **5** and **6** demonstrated high docking scores (Figure 2), making them promising candidates for synthetic targeting.

**Figure 2:**
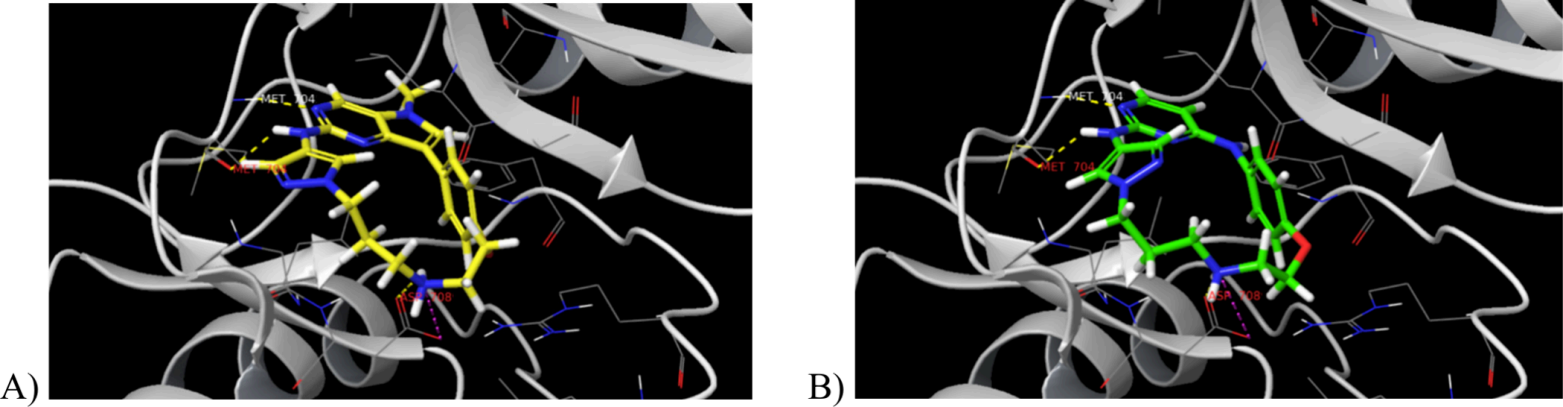
A) Docking pose of compound **5a** in DDR1 (PDB code: 6BRJ). B) Docking pose of compound **6a** in DDR1 (PDB code: 6BRJ). For the docking poses of compounds **5b** and **6b**, see the Supporting Information.

We methylated the pyrrolyl nitrogen in pyrrolopyrimidine cores to ease the synthesis of designed compounds, avoiding potential issues in Pd-catalyzed cross-coupling reactions (Figure 1B). We introduced two distinct moieties into the linker, an amine and an amide, to get four different couples of matched molecular pairs (MMPs).^17^ The selection of these moieties had the goal of enhancing solubility either by introducing positively charged groups (basic secondary amines) or by increasing the polar surface area (incorporation of amidic groups). We obtained therefore **5a** and **5b**, a first pair with the pyrrolopyrimidine core, and **6a** and **6b**, a second MMP with the amino-pyrimidine scaffold (Figure 1B). Interestingly, pairs **5a** **/6a** and **5b** **/6b** can also be seen as MMPs, thus broadening the scope of analysis regarding the impact of structural modification effects.

All macrocyclic MMPs were submitted to computational studies to predict their molecular properties and verify whether they would show an improved profile compared to **1**. The *in silico* study involved a two-step strategy. The first step was the calculation of 2D descriptors (Table S1 and Figure 3).^6^

**Figure 3:**
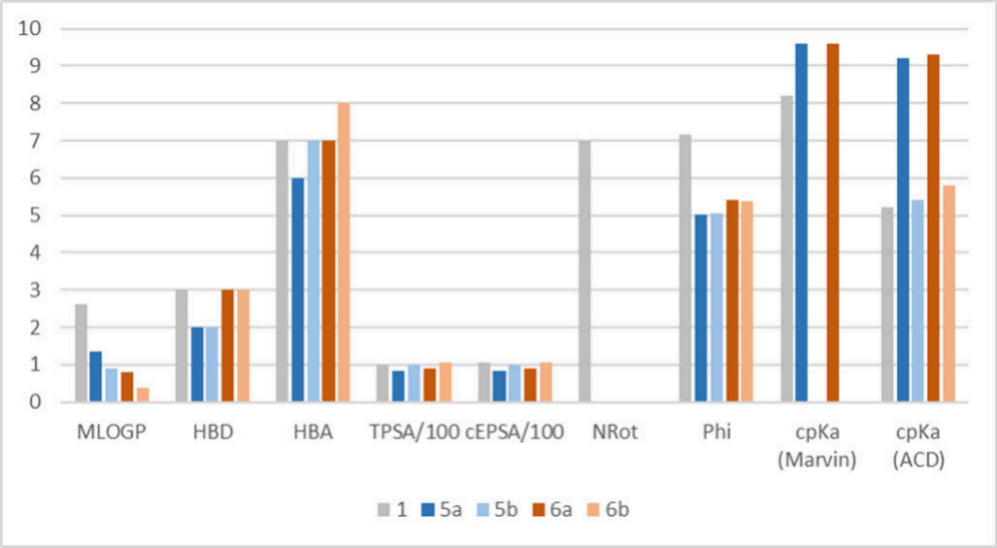
Histograms showing 2D molecular descriptor variation included and not included in the common rules of thumb. MLOGP = lipophilicity index; HBD = number of hydrogen bond donor groups; HBA = number of hydrogen bond acceptor groups; TPSA/100 = topological polar surface area divided by 100; cEPSA = calculated EPSA divided by 100; NRot = number of rotational bonds; Phi = Kier’s flexibility index; cp*K*a (Marvin) = p*K*_a_ calculated with Marvin Sketch; cp*K*_a_ (ACD) = p*K*_a_ calculated with ACD/Labs.

All compounds are Ro5 compliant. The MLOGP (a lipophilicity descriptor not taking ionization into account) of **1** is 2.5 but decreases for cyclized analogues. The number of hydrogen bond donor (HBD) and acceptor (HBA) groups, the topological polar surface area (TPSA), and the calculated EPSA (cEPSA, a polarity descriptor calculated with an internal tool, see SI) do not vary substantially between **1** and the MC derivatives. As expected, flexibility, expressed with the Phi parameter, decreases when introducing cyclization, and **6a** and **6b** are slightly more flexible than **5a** and **5b**. Notably, to describe MCs’ flexibility, Phi should be preferred over the number of rotatable bonds (NRot),^18^ as NRot only accounts for the flexibility of chains attached to the cyclic core, whereas Phi provides a more comprehensive measure of the overall molecular flexibility. p*K*_a_ values were calculated using two widely known tools, Marvin and ACD/Labs. The agreement between them is not optimal. Marvin Sketch seems to overestimate the basicity of **1**. Each software predicts the amine derivatives **5a** and **6a** as predominantly protonated at pH = 7 whereas the amidic derivatives **5b** and **6b** are not expected to show relevant basic properties.

Overall, this *in silico* analysis predicts that the primary remarkable improvement of KIMCs over **1**, from a physicochemical perspective, is attributed to the basicity of the amino group present in **5a** and **6a**. These compounds are expected to be at least partially ionized, thereby enhancing their solubility at physiological pH. No further conclusions can be drawn to prioritize the synthesis of compounds based on 2D descriptor calculations.

The second part of the computational study was focused on predicting the chameleonic behavior^6^ of the investigated derivatives through the calculation and analysis of Rgyr/​3D‑PSA on conformers obtained in polar and nonpolar media. A molecular chameleon tends to display folded and less polar conformations in nonpolar solvents and more open and polar conformations in polar solvents. We sought to determine chameleonicity since it is often regarded as an advantageous property because it can enhance a compound’s ability to solubilize in physiological fluids and cross membranes.^19^ To predict chameleonicity, we submitted the investigated compounds to two conformational sampling (CS) runs in water and in chloroform. Then, for each conformer we calculated polarity (3D-PSA, the lower, the less polar) and sphericity (Rgyr, the lower, the more spherical). Figure 4 shows the polarity vs sphericity plot. Notably, all the MCs but **5b** have at least a few conformers with a polarity comparable to TPSA (red line), i.e., have some completely extended conformers.^20^ Conversely, **5b** has no conformers with 3D-PSA close to TPSA; thus, it is expected to have predominantly folded conformers in both media, so poor chameleonic properties. Overall, this result supports that, chameleonicity being always a positive characteristic to improve drug-like properties, **5a**, **6a**, and **6b** are expected to be better candidates than **5b**.

**Figure 4:**
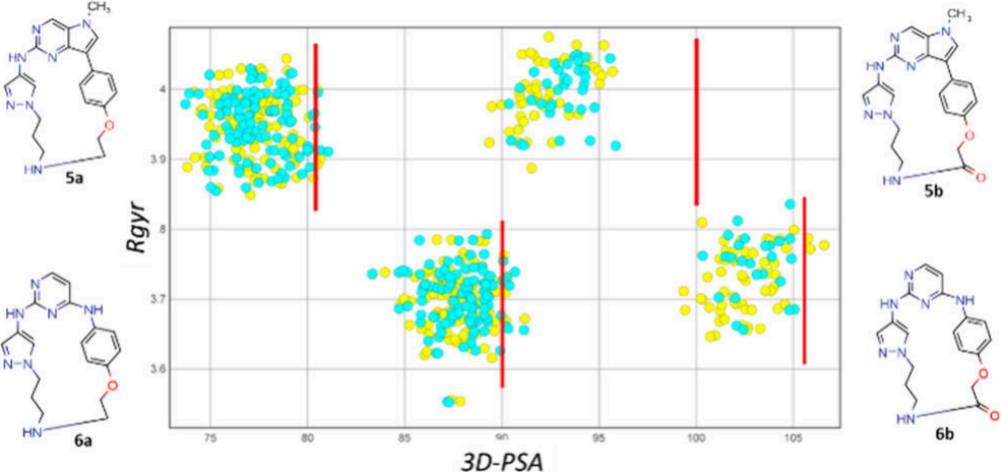
Property analysis of MC conformers in polar (water, turquoise dots) and nonpolar (chloroform, yellow dots) media. Red line = TPSA.

KIMCs **5a**, **5b**, **6a**, and **6b** were then synthesized following the synthetic pathway reported in the SI and profiled *in vitro* (Table 1).

In a U2OS cellular model of DDR1 inhibition (see SI), all compounds proved to be 40- to 1000-fold less potent than **1**. Notably, **5a** was the most active, with an IC_50_ of 125 nM, while the other three exhibited activity in the single-digit micromolar range. Macrocyclization therefore had a detrimental impact on the biological activity of our scaffolds that had not been captured by docking results.

The investigated compounds were then submitted to kinetic solubility and permeability measurements. The uncyclized **1** is almost insoluble, **6a** and **6b** (the flexible pair) are highly soluble and moderately permeable, whereas the two more rigid KIMCs (**5a** and **5b**) are less soluble and highly permeable in the MDCK model. All demonstrated the ability to cross cell membranes in the more sophisticated surrogate of intestinal absorption, the Caco-2 model. Remarkably, in the absence of a P-glycoprotein (Pgp) inhibitor, permeability decreased for **5b**, **6a**, and **6b**. Conversely, **5a** is not a substrate for Pgp.

Stability in human microsomes was also determined. Clearance rates were high for pyrrolopyrimidines, while the stability for **6a** and **6b** was in line with that of compound **1**. We hypothesized that the additional methyl group on the pyrrole ring may have contributed to higher clearance, likely undergoing dealkylation during first-pass metabolism.

Overall, *in vitro* ADME data showed that higher solubilities and superior ADME profiles for KIMCs have been achieved when compared to the linear compound. Moreover, a different trend was found for different MMPs: flexible KIMCs were more soluble and less permeable than more rigid MCs. Interestingly, the decoration on the linker (i.e., the ionization) had a negligible impact, as the difference between the amine and amide MMPs was minimal, contrary to our initial speculation based on 2D predictions. Furthermore, **5a** emerged as the most appealing MC, displaying nanomolar range activity, improved solubility, excellent permeability, and no active transport through cell membranes. Tracing these findings back through preliminary 3D descriptor calculations, compound **5a** exhibited the highest Rgyr and the lowest 3D-PSA (Figure 4), in addition to exhibiting chameleonic behavior.

We finally determined a complete pool of experimental physicochemical descriptors.^21^ First, we measured the p*K*_a_ of **5a**. Two p*K*_a_ values were obtained (see SI): 8.20 (basic) for the amino group and 4.35 (basic) for the pyrrolo­pyrimidine moiety. Although the calculated values overestimate basicity, the experimental determination confirms that this KIMC is mostly protonated at physiological pH. To account for environment-dependent ionization, we also applied a recently developed method to evaluate ionization in nonpolar media.^22^ Plots in Figure S3 support that all KIMCs at pH = 7.0 are predominantly neutral in a nonpolar environment. Then, we measured lipophilicity, polarity, and chameleonicity descriptors (Table 2).

**Table 2:**
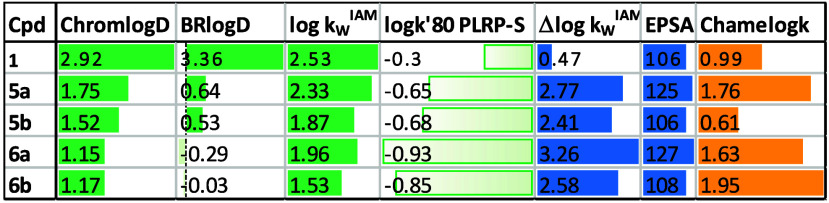
Experimental Physicochemical Descriptors for the Investigated Compounds (See Text for More Details)^a^

a Lipophilicity descriptors: ChromlogD, BRlogD, log k_w_^IAM^, log k′ 80 PLRP-S. Polarity descriptors: Δlog k_w_^IAM^, EPSA. Chamaleonicity descriptor: Chamelogk, >0.6 = chameleons, <0.6 = non-chamaleons.

Lipophilicity has been obtained in four different systems. BRlogD^23^ and ChromlogD^24^ are chromatographic surrogates of log D_oct_ and, despite showing different values, are linearly correlated (Figure S4). Log k′ 80 PLRP-S is a surrogate of log D_tol_.^22^ Log k_w_^IAM^ ^23^ mimics the interaction between the compounds and the phospholipids of membranes. The combination of the experimental lipophilicity descriptors is essential to evaluate lipophilicity in different regions of the membrane bilayer. This complete lipophilicity profile may give an idea of the permeability potential of poorly soluble compounds for which standard permeability measurements are unreliable. For instance, experimental data (Table 2) show that **1** is lipophilic in any system and support its capability to permeate membranes that cannot be assessed because of the low solubility. This is supported by its low IC_50_ value in the cell-based assay. Overall, KIMCs’ lipophilicity is significantly lower than that of the uncyclized derivative **1** in all the systems. This finding correlates with the higher solubility in aqueous media exhibited by cyclized compounds.

Polarity is quantified by two descriptors: EPSA^25^ and Δlog k_w_^IAM^.^23^ The first is measured in an almost fully nonpolar environment, and the second describes polarity in an aqueous medium. Notably, the polarity of **1** is either lower (when expressed as Δlog k_w_^IAM^) or similar (when expressed by EPSA) than that of the MCs. This is also in line with the higher solubility of KIMCs over **1**.

Finally, chameleonicity was measured with the recently described Chamelogk method (Table 2), where 0.6 is the threshold value to distinguish chameleons from non-chameleons.^13^ Chamelogk indicates a chameleonic behavior for all the KIMCs, but for **5b** Chamelogk is significantly lower than for other compounds. This is in line with the computational prediction (Figure 4) and indicates that **5b** has almost negligible chameleonic properties.

According to our experimental evidence,^13^ a compound with BRlogD < 2 and Δlog k_w_^IAM^ > 1.5 has a non-ideal lipophilicity/polarity profile. However, compounds with strong chameleonic properties may compensate for these values. Notably, all the MCs described in this work need chameleonic help to reach an acceptable lipophilicity/polarity profile. Therefore, **5a**, **6a**, and **6b** are expected to have a better lipophilicity/polarity profile than **5b**. In particular, **5a** is the best one because of its highest lipophilicity and comparable polarity.

Physicochemical data revealed key differences between MC pairs. The flexible pair (**6a** and **6b**) exhibits lower lipophilicity and higher polarity than the rigid pair (**5a** and **5b**), indicating greater solubility, as shown in Table 1. The modest variation in lipophilicity and polarity between amidic (neutral) and amine (protonated) derivatives explains the similar solubility and permeability within each pair. These findings suggest that ionization has little impact on this compound class, and core structure changes are more significant than linker design. Such insights would be missed without experimental physicochemical measurements

In this work, we developed macrocyclic alternatives of a linear kinase inhibitor with improved solubility and permeability. To do that we applied two tactics that led to matched pairs differing both in flexibility skills and in the structure of the linking moieties. Pairs analysis provided insights into structure–property relationships.

To evaluate whether the synthesis of KIMCs could lead to derivatives with improved *in vitro* ADME profiles, a molecular property *in silico* study was first performed. Computations based on the 2D structures of macrocyclic derivatives did not support the superior physicochemical and, thus, solubility/permeability profile of the two pairs of KIMCs over the linear lead.

The cyclization process resulted in compounds with reduced activity against the biological target of interest. Nevertheless, we successfully accomplished the primary goal of this design strategy: the development of macrocyclic kinase inhibitors with improved overall properties as alternatives to a linear lead that initially had an unfavorable developability profile. Notably, compound **5a** demonstrated nanomolar potency and emerged as a highly promising cyclic candidate. We also showed that the chameleonic behavior is expected to be at least in part responsible for the improved MC physicochemical profile.

As a lead-like compound, **5a** is well-positioned for further optimization efforts aimed at improving its potency while preserving its already favorable developability characteristics.

## Safety

The authors declare that no major criticalities were identified for the reactions reported in this paper, based on our chemical risk assessment.

## Supplementary Material



## Notes

Tables, figures, methods (computational, physicochemical, *in vitro* ADME, and *in vitro* biology), and chemistry (material, docking studies, synthesis, analytical methods, structural characterization, purity and NMR spectra of macrocycles) (PDF)

## Abbreviations

3D-PSA: three-dimensional polar surface area
ADME: absorption, distribution, metabolism, and excretion
Caco-2: human colorectal adenocarcinoma cells
DDR: discoidin domain receptor
ECM: extracellular matrix
EPSA: experimental polar surface area
KIMC: kinase inhibitor macrocycle
KS: kinetic solubility
MC: macrocycles
MDCK: Madin–Darby canine kidney
MMP: matched molecular pair
Pgp: P-glycoprotein
TPSA: topological polar surface area

## References

[1] Olaso, E. ; Marquez, J. ; Benedicto, A. ; Badiola, I. ; Arteta, B. Discoidin Domain Receptors in Liver Fibrosis. In Discoidin Domain Receptors in Health and Disease; Fridman, R. , Huang, P. H. , Eds.; Springer: New York, 2016; pp 293–313. 10.1007/978-1-4939-6383-6_16.

[2] Dorison A., Dussaule J. C., Chatziantoniou C. (2017). The Role of
Discoidin Domain Receptor
1 in Inflammation, Fibrosis and Renal Disease. Nephron.

[3] Moll S., Desmoulière A., Moeller M. J., Pache J. C., Badi L., Arcadu F., Richter H., Satz A., Uhles S., Cavalli A., Drawnel F., Scapozza L., Prunotto M. (2019). DDR1 Role
in Fibrosis and Its Pharmacological Targeting. Biochim. Biophys. Acta, Mol. Cell Res..

[4] Jia S., Agarwal M., Yang J., Horowitz J. C., White E. S., Kim K. K. (2018). Discoidin Domain
Receptor 2 Signaling Regulates Fibroblast
Apoptosis through PDK1/Akt. Am. J. Mol. Biol..

[5] Driggers E. M., Hale S. P., Lee J., Terrett N. K. (2008). The Exploration
of Macrocycles for Drug Discovery - An Underexploited Structural Class. Nat. Rev. Drug Discovery.

[6] Garcia
Jimenez D., Poongavanam V., Kihlberg J. (2023). Macrocycles in Drug
Discovery—Learning from the Past for the Future. J. Med. Chem..

[7] Blanco M. J., Gardinier K. M. (2020). New Chemical
Modalities and Strategic Thinking in Early
Drug Discovery. ACS Med. Chem. Lett..

[8] Blanco M. J., Gardinier K. M., Namchuk M. N. (2022). Advancing New Chemical Modalities
into Clinical Studies. ACS Med. Chem. Lett..

[9] Liang Y., Fang R., Rao Q. (2022). An Insight into the Medicinal Chemistry
Perspective of Macrocyclic Derivatives with Antitumor Activity: A
Systematic Review. Molecules.

[10] Mallinson J., Collins I. (2012). Macrocycles in New
Drug Discovery. Future Med. Chem..

[11] Johnson T.
W., Richardson P. F., Bailey S., Brooun A., Burke B. J., Collins M. R., Cui J. J., Deal J. G., Deng Y. L., Dinh D., Engstrom L. D., He M., Hoffman J., Hoffman R. L., Huang Q., Kania R. S., Kath J. C., Lam H., Lam J. L., Le P. T., Lingardo L., Liu W., McTigue M., Palmer C. L., Sach N. W., Smeal T., Smith G. L., Stewart A. E., Timofeevski S., Zhu H., Zhu J., Zou H. Y., Edwards M. P. (2014). Discovery of (10
R)-7-Amino-12-Fluoro-2,10,16-Trimethyl-15-Oxo-10,15,16,17- Tetrahydro-
2H −8,4-(Metheno)­Pyrazolo­[4,3- h]­[2,5,11]- Benzoxadiazacyclotetradecine-3-Carbonitrile
(PF-06463922), a Macrocyclic Inhibitor of Anaplastic Lymphoma Kinase
(ALK) and c-Ros Oncogene 1 (ROS1) with Preclinical Brain Exposure
and Broad-Spectrum Potency against ALK-Resistant Mutations. J. Med. Chem..

[12] Landis M. S., Bhattachar S., Yazdanian M., Morrison J. (2018). Commentary: Why Pharmaceutical
Scientists in Early Drug Discovery Are Critical for Influencing the
Design and Selection of Optimal Drug Candidates. AAPS PharmSciTech.

[13] Garcia
Jimenez D., Vallaro M., Rossi Sebastiano M., Apprato G., D’Agostini G., Rossetti P., Ermondi G., Caron G. (2023). Chamelogk: A Chromatographic Chameleonicity Quantifier to Design
Orally Bioavailable Beyond-Rule-of-5 Drugs. J. Med. Chem..

[14] Amrhein J. A., Knapp S., Hanke T. (2021). Synthetic
Opportunities and Challenges
for Macrocyclic Kinase Inhibitors. J. Med. Chem..

[15] De S. K. (2023). First Approval
of Pacritinib as a Selective Janus Associated Kinase-2 Inhibitor for
the Treatment of Patients with Myelofibrosis. Anti-Cancer Agents Med. Chem..

[16] Ma J., Sanchez-Duffhues G., Caradec J., Benderitter P., Hoflack J., ten Dijke P. (2022). Development
of Small Macrocyclic
Kinase Inhibitors. Future Med. Chem..

[17] Leach A. G., Jones H. D., Cosgrove D. A., Kenny P. W., Ruston L., MacFaul P., Wood J. M., Colclough N., Law B. (2006). Matched Molecular Pairs as a Guide in the Optimization of Pharmaceutical
Properties; a Study of Aqueous Solubility, Plasma Protein Binding
and Oral Exposure. J. Med. Chem..

[18] Caron G., Digiesi V., Solaro S., Ermondi G. (2020). Flexibility in Early
Drug Discovery: Focus on the beyond-Rule-of-5 Chemical Space. Drug Discovery Today.

[19] Poongavanam V., Wieske L. H. E., Peintner S., Erdélyi M., Kihlberg J. (2024). Molecular Chameleons in Drug Discovery. Nat. Rev. Chem..

[20] Rossi
Sebastiano M., Garcia Jimenez D., Vallaro M., Caron G., Ermondi G. (2022). Refinement of Computational Access to Molecular Physicochemical
Properties: From Ro5 to BRo5. J. Med. Chem..

[21] Ermondi G., Garcia Jimenez D., Rossi Sebastiano M., Caron G. (2021). Rational Control of
Molecular Properties Is Mandatory to Exploit the Potential of PROTACs
as Oral Drugs. ACS Med. Chem. Lett..

[22] Caron G., Vallaro M., Ermondi G., Goetz G. H., Abramov Y. A., Philippe L., Shalaeva M. (2016). A Fast Chromatographic Method for
Estimating Lipophilicity and Ionization in Nonpolar Membrane-Like
Environment. Mol. Pharmaceutics.

[23] Ermondi G., Vallaro M., Goetz G., Shalaeva M., Caron G. (2020). Updating the
Portfolio of Physicochemical Descriptors Related to Permeability in
the beyond the Rule of 5 Chemical Space. Eur.
J. Pharm. Sci..

[24] Valkó K., Bevan C., Reynolds D. (1997). Chromatographic Hydrophobicity Index
by Fast-Gradient RP-HPLC: A High-Throughput Alternative to Log P/Log
D. Anal. Chem..

[25] Goetz G. H., Farrell W., Shalaeva M., Sciabola S., Anderson D., Yan J., Philippe L., Shapiro M. J. (2014). High Throughput Method for the Indirect
Detection of Intramolecular Hydrogen Bonding. J. Med. Chem..

