# Supplementary material for: Macrocyclization: Enhancing Drug-like Properties of Discoidin Domain Receptor Kinase Inhibitors
Source: ACS Med Chem Lett. 2025 Apr 7;16(5):784–9. doi: 10.1021/acsmedchemlett.4c00611 (PMC12067128; doi:10.1021/acsmedchemlett.4c00611)
Supplement: Supplementary file 1 [file ml4c00611_si_002.pdf]

Supporting Information

Macrocyclization: Enhancing Drug-Like Properties of DDR Kinase Inhibitors

Laura Carzaniga[#], Roberta Mazzucato[#], Valentina Mileo[#], Andrea Rizzi[#], Maura Vallaro[&], Giuseppe Ermondi[&], Silvia Cattani[@], Andrea Secchi[@] and Giulia Caron[*&]*

[#] Chiesi Farmaceutici, Corporate Preclinical R&D, Research Center, Largo Belloli 11/A, 43122, Parma, Italy

[&]University of Torino, Molecular Biotechnology and Health Sciences Dept., CASSMedChem, Piazza Nizza 44bis, 10126 Torino, Italy.

[@]University of Parma, Department of Chemistry, Life Sciences and Environmental Sustainability, Parco Area delle Scienze 17/A, 43123 Parma

Corresponding author*: Giulia Caron and Laura Carzaniga

Table of contents

Tables

Table S1. Common 2D molecular descriptors included and not included in the common rules of thumb.

*Table S2. pK_a values of **5a***

Figures

Figure S1. Selected MKIs approved or under evaluation in clinical trials.

Figure S2 Docking pose of compound 5b in DDR1 (PDB code:6BRJ) B) Docking pose of compound 6b in DDR1 (PDB code:6BRJ).

Figure S3. Ionization behavior of the investigated compounds as obtained by monitoring the variation of the log of the capacity factor in the PLRP-S system (log k'80 PLRP-S) vs the pH.

Figure S4. Lipophilicity: two chromatographic surrogates of log Doct are linearly correlated

Figure S5. Polarity: relationship between EPSA and Dlog kwIAM

Computational methods

Docking Results for 1, 2, 3 ,4, longer and shorter linker length analogues

Physicochemical methods

In vitro Biology methods

In vitro ADME methods

Chemistry

Materials

Synthesis

Analytical methods

Summary of structural characterization and purity of macrocycles

NMR spectra

References

Tables

***Table S1**. Common 2D molecular descriptors included and not included in the common rules of thumb*

	**1**	**5a**	**5b**	**6a**	**6b**
**Pair**	-	rigid	rigid	flexible	flexible
**Group**	-	amine	amide	amine	amide
**MW**	491	389	403	351	365
**MLOGP**	2.63	1.35	0.91	0.81	0.37
**nHDon**	3	2	2	3	3
**nHAcc**	7	6	7	7	8
**TPSA**	101	82	99	89	106
**cEPSA**	107	82	99	89	106
**NRot**	7	0	0	0	0
**Phi**		5.02	5.04	5.40	5.39
**cpK_a (Marvin)**	8.2	9.6	*Not ionizable*	9.6	*Not ionizable*
**cpK_a (ACD)**	5.2	9.2	5.4	9.3	5.8

Table S2**. pK_a values of **5a

**Mean value[a]**	**SD**	**Tentative site attribution**	**Solvent**
8.20	0.02	Propan-1-amine	MeOH:ISA water
4.35	0.04	Pyrrolo-pyrimidine	MeOH:ISA water

[a]Reported are mean values ± SD (n=4).

Figures

***Figure S1**. Selected MKIs approved or under evaluation in clinical trials.*

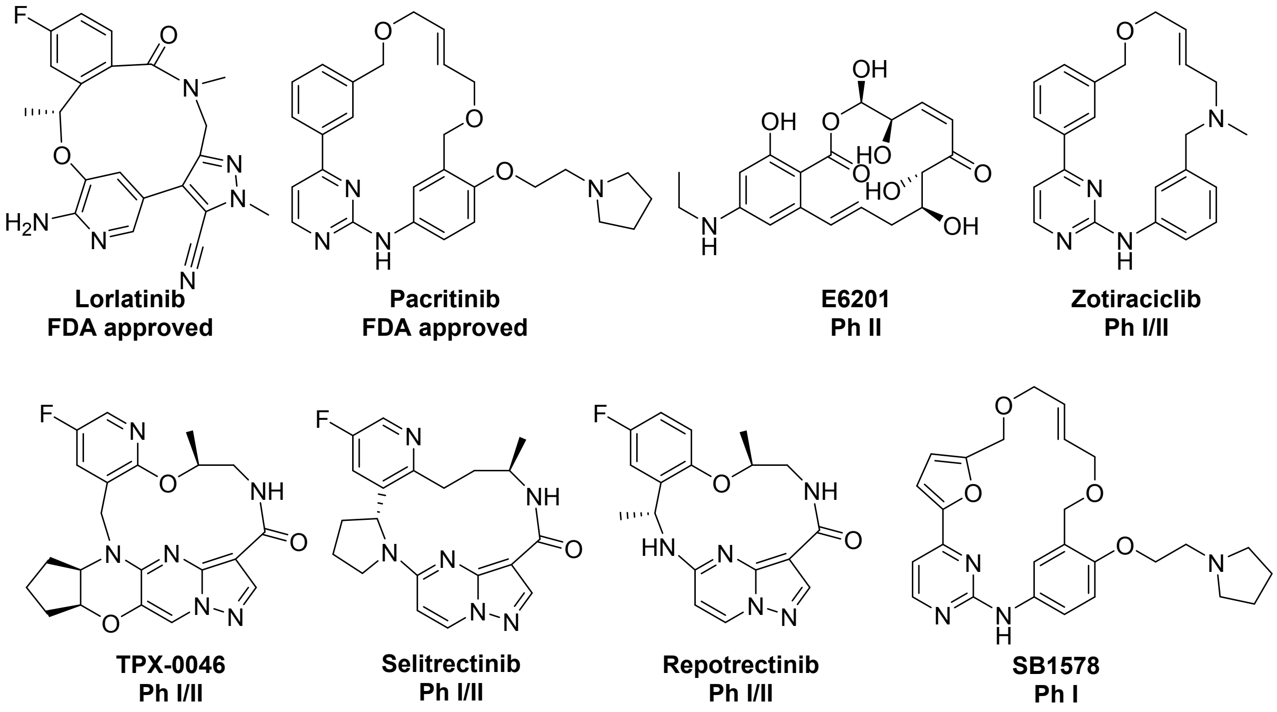

Figure S2 *Docking pose of compound* ***5b*** *in DDR1 (PDB code:6BRJ) B) Docking pose of compound* ***6b*** *in DDR1 (PDB code:6BRJ).*

A) 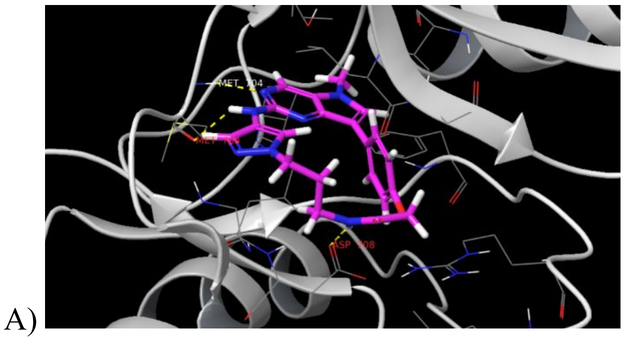 B) 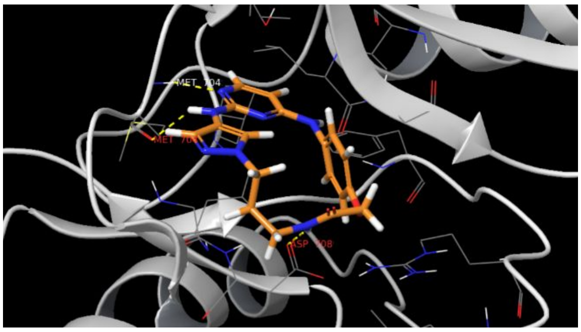

Figure S3. *Ionization behavior of the investigated compounds as obtained by monitoring the variation of the log of the capacity factor in the PLRP-S system (log k'80 PLRP-S) vs the pH.*

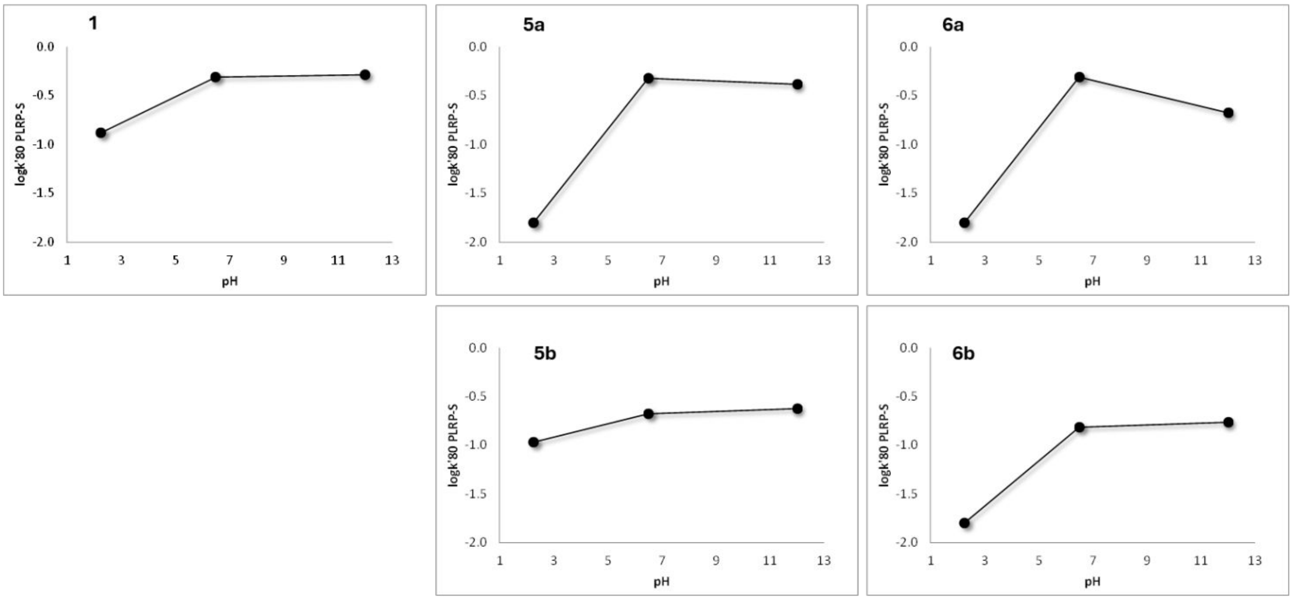

Figure S4. *Lipophilicity: two chromatographic surrogates of log Doct are linearly correlated*

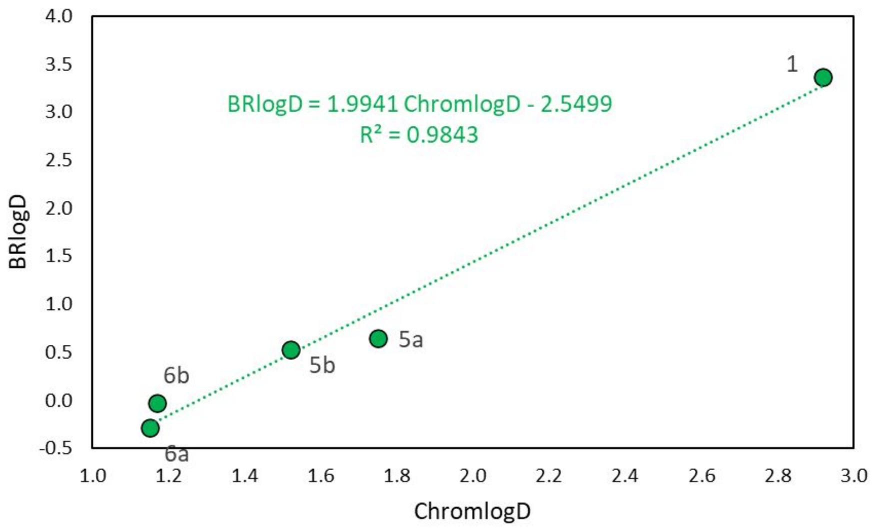

Figure S5. *Polarity: relationship between EPSA and ∆log kwIAM*

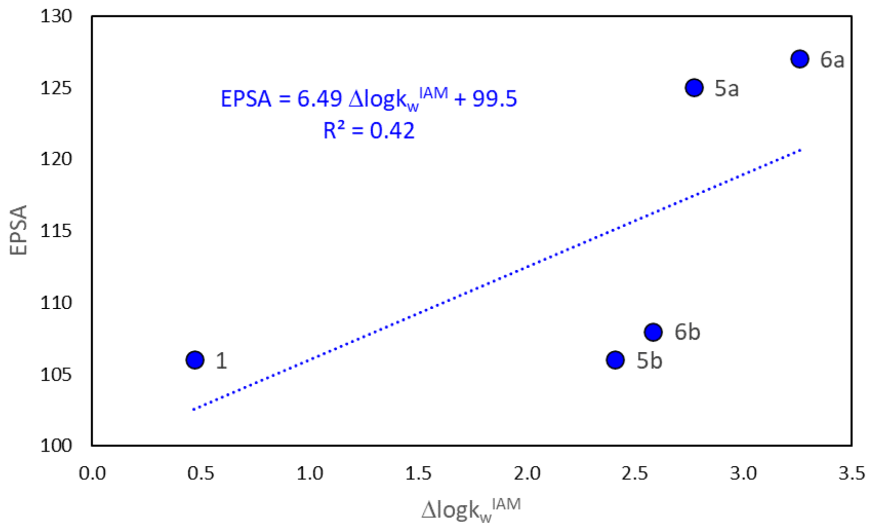

Computational Methods

2D descriptors

The SMILES codes were submitted to SwissADME (http://www.swissadme.ch/), Marvin JS (v. 24.3.0, https://playground.calculators.cxn.io/), ACD/Labs v2023 (https://www.acdlabs.com/) and AlvaDesc v.2.016 (https://www.alvascience.com/) that allowed 2D descriptors calculation.

cEPSA

The molecules are featurized using RdKit. This involves extracting both atom features (such as atom type, degree, valence, hybridization state, etc.) and bond features (bond type, conjugation, etc.). Subsequently, these features undergo processing using a Message Passing Neural Network model.[a] To enhance the propagation of global information during the forward pass, the molecular graph is augmented by introducing a virtual node connected to all the atoms within the molecule. An Edge Network[a] is employed to act as a messenger. In terms of aggregation, a straightforward summation approach is used. Activations are updated using a gated recurrent unit. In the final step, a sequence-to-sequence readout function[b] is utilized to derive a vector of neural fingerprints. These fingerprints are then further processed through a sequence of fully connected layers, and a final linear layer generates multiple outputs corresponding to various tasks. For training purposes, we employ publicly available experimental data from ChEMBL, in addition to our proprietary dataset encompassing physical-chemical, molecular stability, and binding properties. The training process follows a supervised approach, employing an ADAM optimizer.[c] During training, the loss is calculated based on the available experimental values for each molecule. The multitask nature of the model and the training procedure facilitate the capture of correlations between different output values and the development of features relevant to multiple tasks, thereby introducing a form of regularization.

Docking

A preliminary binding pose of 5a in the catalytic domain of DDR1 was obtained applying the standard precision (SP) docking protocol in Glide. The resulting protein-ligand complex was embedded in an orthorhombic TIP3P water box and a molecular dynamics (MD) simulation was performed using Desmond (v. 7.7, Schrödinger, LLC, New York, NY). After a simulation of 100 ns a representative frame was extracted and was minimized in Prime (v. 7.5, Schrödinger, LLC, New York, NY). This refined protein structure was used as reference docking grid for subsequent docking of other macrocycle ligands in Glide (v. 10.6 - Schrödinger, LLC, New York, NY) applying the SP method.

Chameleonicity prediction

The MacroModel plugin in the Maestro suite (Schrödinger, https://newsite.schrodinger.com/) was applied to obtain conformational sampling as reported elsewhere.[d] The calculation of 3D PSA and Rgyr was performed in VEGA ZZ (http://www.vegazz.net/) by importing the conformer structures as unique files in mol2 format. All the descriptors from Vega ZZ were calculated with standard settings. Specifically, 3D PSA had a probe radius with the default value (0).

In water, 114 and 37 conformations were obtained for **5a** and **5b** respectively, and 110 and 25 for **6a** and **6b**, respectively. In chloroform, 114 and 51 for **5a** and **5b** respectively, and 110 and 59 for **6a** and **6b**, respectively.

Docking Results for 1, 2, 3 ,4, longer and shorter linker length analogues All macrocycles were prioritized after visual analysis of the docked poses and after assessment of Glide Emodel scores.

The docking protocol was applied on an optimized DDR1 model (PDB code: 6BRJ), where the protein kinase binds the ligands a DFG-in conformation.

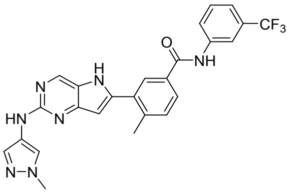

1

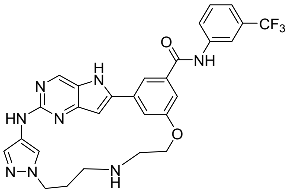

2

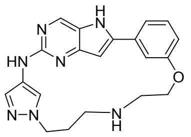

3

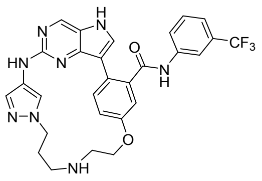

4

6 (score: -58.383) and **5** (score: -114.781) demonstrated to be the most promising macrocycle compounds in comparison to **2** (score: -9.640), **3** (score: -12.225), and **4** (score: -25.485).

1 did not show a correct binding mode since it is a potent inhibitor designed to bind a DFG-out conformation of a kinase protein.

Docking in 3ZOS DDR1 protein (DFG-out conformation) confirmed that **1** is an excellent binder (score: -125.767) and demonstrated that all macrocycle structures are unable to bind in an efficient way this specific protein conformation.

The most appropriate length of the linker was checked applying ligand-receptor docking in 6BRJ protein model.

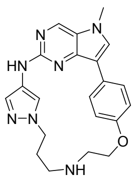

5a (3+2)

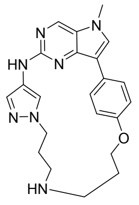

3+3

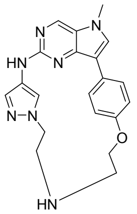

2+2

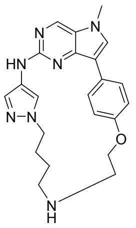

4+2

The reference macrocycle **3+2** received a Glide Emodel of -114.781. The elongation and the shortening of the linker between the pyrazole ring and the protonated amino group generated analogues with worse scores: **4+2** (score: -50.498) and **2+2** (score: -64.535). Similarly, the extension of the linker between the phenol fragment and the protonated amino group worsened, albeit to a lesser extent, the docking results: **3+3** (score: -94.237).

Physicochemical Methods

pH-metric determination of pK_a values

Experimental pK_a was determined by pH-metric approaches using SiriusT3 (PIon Inc. Ltd, Forrest Row, UK) instrument which integrates a dispenser module equipped with precision dispensers, 0.5 mL syringes, a multi-tip capillary bundle, a titrator module, a microstirrer with variable speed, a combined double junction Ag/AgCl pH and reference electrode and a fibre optics dip probe connected to a deuterium UV pulsed light source and a photodiode array (PDA) detector, acquiring spectra in the 200-700 nm wavelength range. SiriusT3 Control software v 1.1.3.0 was employed for acquisition, while Sirius T3 Refine software v. 1.1.3.0 was used for data analysis. The glass microelectrode was standardized by means of a strong acid-strong base titration (0.5N HCl vs. 0.5N KOH) in the pH range 2.0-12.0 at fixed temperature (25±0.5°C) and I = 0.15 M for KCl addition, in N_2 atmosphere. The blank titration allowed to obtain the electrode "Four plus parameters" which enables to convert, by means of a multi-parametric equation, the operational pH activity values into concentration values. 0.5 N KOH was standardized employing potassium hydrogen phthalate as primary standard by means of three independent titrations of weighted amounts (approx. 15 mg per assay). The stock solution in DMSO of compound was dissolved at the pH at which it was more soluble (i.e. pH=2.0) by titrant addition before starting the assay. Due to solubility issues in ISA water, a cosolvent/water approach was followed for pK_a determination, and the aqueous pK_a values were extrapolated to 0% cosolvent content by the Yasuda-Shedlovsky approach embedded in Sirius T3 Refine software.

BRlogD

The original method was described in a previous paper.[e] Briefly, a HPLC Thermo Fisher Scientific Dionex Ultimate 3000 instrument equipped with a Low-Pressure Mixing Gradient Pump LPG-3400 SD, ACC-3000 autosampler with termostatable column compartment, a DAD-3000 RS Detector and Chromeleon Data System Version 7.2.10 was used. Deionized water and HPLC grade acetonitrile were used throughout. The mobile phases consisted of 20 mM ammonium acetate at pH 7.0 and acetonitrile 60% v/v. Samples were dissolved in a 1:1 mixture of buffer/acetonitrile in the concentration range of 50–100 ug/mL. A 10uL volume of each solution was injected at 30°C and flow rate was 1 mL/min. The dead time, t0, was determined by monitoring the baseline disturbance. The

retention time (t_R) was measured on a XBridgeTM Shield RP18 (Waters, 5cm x 4.6 mm, 5 µm particle size, 130Å pore size). Isocratic log k' (capacity factor k' = (t_R - t_0)/t_0) values were measured. The relationship between log D and log k'60 has been determined previously and is reported below (Eq. 1)

log D = 3.31*log k'60 + 2.79 *Eq. 1*

We termed this log D as BRlogD.

ChromLogD

The chromatographic method was based on the differential retention times of test compounds on a C18 column (Luna C18(2), 30 x 2mm, 3mm, 100 Å) under controlled liquid chromatography conditions, comparing retention times with those of standards (Benzyl Alcohol, p-Nitrophenol, Bromobenzene, Anthracene) with known log D values. Stock solutions (10 mM in DMSO) of test compounds were diluted to a final concentration of 1 mM. The mobile phases consisted of A (20mM Ammonium Acetate buffered to pH 7.4 with ammonia hydroxide) and B (ACN). The LC gradient profile was the following: a linear increase to 90% B during 0.0 to 1.3 min, hold at 90 % B during 1.3 to 1.53 min then back to 0.0% B from 1.53 to 1.60 min, hold at 0% B until the end of the run (2.00 min). 1 µL of the sample volume was injected, using a flow rate of 0.5 mL/min and a column temperature of 40°C. All compounds were detected by UPLC/MS analysis using a a Waters ACQUITY UPLC equipped with a Photo Diode Array (PDA) detector coupled with a Waters ACQUITY QDa single quadrupole mass spectrometer with an Electrospray ionization (ESI) source. The retention times were obtained by processing data in Waters Openlynx. The standards are then plotted in excel and the best fit line was used as the basis for calculating the unknown LogD values from the retention times of the peaks obtained.

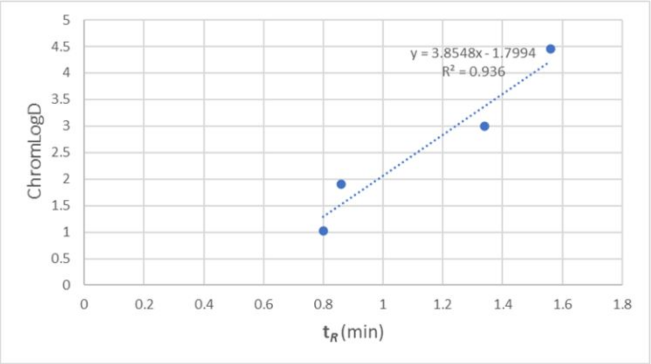

EPSA

EPSA values were measured following the SFC protocol set up by Goetz and co-workers.[f] This method implies the use of the Pirkle chiral stationary phase Chirex 3014, a silica bonded (S)-valine and (R)-1-(α-naphthyl)-ethylamine with a urea linkage, and a mobile phase of supercritical CO_2 with the addition of 20 mM ammonium formate in methanol as a modifier at a flow rate of 5 mL/min. The modifier was increased from 5 to 60% in 11 min at 5%/min in a linear gradient, keeping at 60% for 4.9 min and coming back to the original 5% in 0.1 min. Samples were dissolved in DMSO, and the injection volume was 5 µL. The column temperature was set to 40°C. Each sample was analyzed in duplicate. A daily-generated linear relationship between retention time and the assigned EPSA of eight standards (antipyrine, chlorpromazine, desipramine, pindolol, diclofenac, 3-nitrobenzoic acid, bumetanide and furosemide) was used to calculate EPSA values. EPSA analyses were carried out using supercritical fluid chromatographer (SFC; JASCO SFC-4000, Jasco Europe srl), equipped with a diode array and operating with ChromNAV 2.04.00 software (www.jascoweb.com).

$\Delta logk_w^{IAM}$

The applied method to measure log k^w_{IAM} was already described elsewhere.[e] Briefly, the analyses were performed at 30°C with 20 mM ammonium acetate at pH 7.0 in mixture with acetonitrile at various percentages. The stationary phase was IAM.PC.DD.2 (Regis Technology, 10 cm × 4.6 cm 10 µm packing 300 Å pore size). The flow rate was 1.0 mL/min, and the injection volume was 10 µL. Chromatographic retention data at a given amount of cosolvent, expressed as log k_{IAM} (the logarithm of the retention factor), were calculated by the expression:

$$\log k_{IAM} = \log [(t_R - t_0)/t_0] \qquad \textit{Eq. 2}$$

where t_R and t_0 are the retention times of the drug and a non-retained compound (citric acid), respectively. All log k_{IAM} values are the average of at least three measurements. The indexes log k^w_{IAM} were calculated by an extrapolation method.

Chamelogk

The method has been described in a former paper.[g] Briefly, a PLRP-S column (100 Å, 5µm, 50 × 4.6 mm; from Agilent (www.agilent.com)) was used, flow rate 1 mL/min, and controlled temperature of 30°C. Isocratic log k' (capacity factor k' = $(t_R - t_0)/t_0$) values were measured, where (t_R) was the

retention time, and t_0, the dead time, was determined by monitoring the baseline disturbance. The first step involves the experimental measurement of log k'PLRP-S values at 50, 60, and 70% of MeCN. A linear fitting between log k'PLRP-S and the % MeCN can be obtained with an expected high R^2 ($R^2 \geq 0.90$). This linear regression is used to obtain an extrapolated log k'PLRP-S value at 100% MeCN (named Ext. log k'100). Finally, we experimentally measure the log k'PLRP-S value at 100% MeCN (named Exp. Log k'100). We defined Chamelogk as the capacity factor difference between the experimental log k measured with 100% MeCN (Exp. Log k'100) and the extrapolated correspondent value (Ext. log k'100), as reflected by the equation:

Chamelogk = Exp. log k'100-Ext. log k'100 *Eq. 3*

In vitro Biology

DDR1 cellular assay

The activity of Discoidin Domain-containing Receptor protein kinase (DDR) inhibitors was determined using the PathHunter® U2OS DDR1 Functional Assay which employs Enzyme Fragment Complementation (EFC) technology, where the β-galactosidase (β-gal) enzyme is split into two fragments, ProLink (PK) and Enzyme Acceptor (EA). Independently these fragments have no β gal activity; however, when forced to complement through protein-protein interactions, they form an active β-gal enzyme. In the PathHunter® U2OS DDR1 Functional Assay, the U2OS human osteosarcoma cell line is modified to co-express a ProLink™ (PK) tagged full-length DDR1b and an Enzyme Acceptor (EA) tagged SH2 domain. Collagen II, as a ligand, induces activation of DDR1b which results in cross-phosphorylation. The SH2-EA fusion protein then specifically binds the phosphorylated receptor resulting in complementation of the two fragments of β-gal and formation of a functional enzyme. β-gal activity is then quantitatively detected using a chemiluminescent substrate.

PathHunter® Human U2OS DDR1 cells were prepared in AssayComplete™ Cell Plating 16 Reagent (DiscoverX 93-0563R16A) and plated at 5000 cells/well in a 384-well white, clear-bottom, sterile plate (ThermoFisher 142762) and incubated for 90 minutes at 37 °C, 5% CO2. Test compounds were serially diluted from a top concentration of 10 mM (1000x final assay concentration) directly in a 384-well low-volume polypropylene plate (Greiner bio-one 784201) using an Echo 555 acoustic liquid handler (Labcyte). Test compounds were diluted in AssayComplete™ Cell Plating 16 Reagent then added to cells using an Integra ViaFlow384 electronic reagent dispenser and pre-incubated at 37 °C, 5% CO2 for 30 minutes. Following compound pre-incubation, the cells were stimulated with 20 µg/ml Bovine Type II Collagen (DiscoverX 92-1090) with incubation for 20 hours at 37 °C, 5% CO2. Next, PathHunter Detection Reagent (DiscoverX 93-0001L) was added to the cells, incubated for 60 minutes at 25 °C in the dark, prior to capturing the chemiluminescent signal on a PHERAstar (BMG LABTECH).

The percentage of inhibition was calculated for each concentration of compound using a 0.1% DMSO vehicle/Collagen II control response as a 0% inhibition and a 10 nM Dasatinib/Collagen II positive

control response as a 100% inhibition. The final assay concentration of the top of the test compound CRC was 10 µM.

In vitro ADME methods

Solubility

Using a stock solution of test compound in 100% DMSO, duplicate dilutions were prepared at a nominal concentration of 200 µM in 0.1 M phosphate buffered saline (PBS) at pH 7.4, with a 2% final DMSO content. Test compound calibration standards were prepared at 200 µM and 10 µM in 100% DMSO. The PBS and DMSO dilutions were equilibrated on a shaking platform at room temperature for two hours prior to filtration using a Multiscreen HTS solubility filter plate (Millipore). Filtrates were analysed by LC-UV-MS and the concentration of compound in PBS filtrate determined by comparing the UV absorbance peak with that of the two DMSO calibration standards. Mass spectrometry will be used to confirm the presence of the expected molecular ion in the UV peak measured. The effective range of the assay is 5 – 200 µM.

Permeability

MDCK-WT permeability assay

MDCK-WT wild-type cells were seeded onto 24-well Transwell plates at 2.35 x 10^5 cells per well and used in confluent monolayers after a 3-day culture at 37°C under 5% CO_2. For all cell types, test and control compounds (propranolol, quinidine) were added (10 µM, 0.1% DMSO final, n=2) to donor compartments of the Transwell plate assembly in assay buffer (Hanks balanced salt solution supplemented with 25 mM HEPES, adjusted to pH 7.4) for both apical to basolateral (A>B) and basolateral to apical (B>A) measurements. Incubations were performed at 37°C, with samples removed from both donor and acceptor chambers at T=0 and 1 hour, and compound analysed by mass spectrometry (LC-MS/MS) including an analytical internal standard.

Apparent permeability (P_{app}) values were determined from the relationship:

$$P_{app}(\times 10^{-6}\ cm/s) = \frac{[Compound]_{receiver} \times V_{receiver} \times 1{,}000{,}000}{[Compound]_{reference} \times T_{inc} \times 60 \times Surface\ Area} \qquad \textit{Eq. 4}$$

Where:

- [compound] = test compound MS peak height ÷ analytical internal standard MS peak height
- V = volume of Transwell compartment (apical = 0.125 mL, basolateral = 0.60 mL)
- T_{inc} = incubation time (60 minutes)
- Surface area = area of cells exposed for drug transfer (0.33 cm^2)

Lucifer Yellow (LY) was added to the apical buffer in all wells to assess viability of the cell layer. As LY cannot freely permeate lipophilic barriers, a high degree of LY transport indicates poor integrity of the cell layer and wells with a LY Papp > 10 x 10^{-6} cm/s were rejected. Compound recovery from the wells was determined from MS responses (normalised to internal standard) in donor and acceptor chambers at the end of incubation compared to response in the donor chamber pre-incubation. Recoveries < 50 % suggest compound solubility, stability or binding issues in the assay which may reduce the reliability of a result.

Caco-2 membrane permeability assay

Caco-2 cells (ECACC) were cultured in DMEM, 10% FBS, 1% NEAA, Hepes buffer 10 mM and in 50 U/mL penicillin and 50 mg/mL streptomycin. For transport studies, 200,000 cells/well were seeded on Millicell 24-well cell culture plates. After 24 hours of incubation at 37 °C and 5% CO_2, the medium was changed with Enterocyte Differentiation Medium (Becton Dickinson), which allows Caco-2 cells to establish within three days a differentiated enterocyte monolayer. The permeability of the compounds across the cells' monolayer was determined by measuring their transport in both directions: apical to basolateral (A → B) and basolateral to apical (B → A). For A → B directional transport, the donor working solution with test article 10 m M) was added to the apical (A) compartment and HBSS (pH 7.4) was added to the basolateral (B). For B → A directional transport, the donor working solution (with test article 10 m M) was added to the basolateral (B) compartment and HBSS (pH 7.4) was added to the apical (A). Samples were collected at T0 and after 2h incubation (5% CO2, 95% relative humidity, 37 °C) for LC-MS/MS analyses. The Papp expressed as nm/sec was calculated as follows: Papp (nm/sec) = (Vr/C0) x (1/A) x (Cr(t)/T) x 1000 000. Where Vr is the volume of the solution in the receiving compartment (mL); C0 is initial test compound concentration in the donor compartment (expressed as area ratio); A is the membrane surface area (cm2); Cr (t) = measured concentration of the receiver well at time T120 (expressed as area ratio) and T is time of incubation (sec). Efflux ratio was also measured to understand if the compounds undergo active efflux: Efflux ratio = Papp [B > A] / Papp [A > B]. The rank order of apparent permeability of the test compound was compared with that of known reference compounds tested in the same experiment, including Sulpiride and Metoprolol (as marker for low and high permeability, respectively and Talinolol (as known P-gp substrate). The general absorption classification for Papp

values in Caco-2 assay were: <10 nm/s = low level; 10-50 nm/s = medium level; >50 nm/s = high level.

Microsomes Stability

Test compounds were incubated, in duplicate, at the concentration of 1 μM with liver microsomes (0.8 mg protein/mL) in Dulbecco's buffer (pH 7.4) at 37 °C in the presence of 1 mM NADPH. At different time points (0, 5, 10, 20, 30, and 60 min), 50 μL aliquots of the incubates were taken, added with 80 μL of ice-cold acetonitrile, and 20 μL of 1 μM warfarin in acetonitrile (injection check) to stop the reaction, and samples were centrifuged. The supernatants were analyzed by LC−MS/MS for unchanged compounds. Test compounds were incubated with liver microsomes in Dulbecco's buffer in the absence of NADPH for 0 and 60 min, as control. Midazolam at the concentration of 1 μM, was incubated with microsomes as positive control for phase I activity of microsomes. Control samples were processed as test compound samples.

Chemistry

Materials

All solvents were dried using standard procedures. All other reagents were of reagent-grade quality, obtained from commercial suppliers and were used without further purification. "Brine" refers to a saturated aqueous solution of NaCl. Unless otherwise specified, solutions of common inorganic salts used in workups are aqueous solutions. Reactions were monitored by TLC using 0.25 mm Merck silica gel plates (60 F254) or LC–MS analysis. Automated column chromatography purifications were done using a Biotage Isolera apparatus with prepacked silica gel or C18 columns of different sizes (from 10 to 120 g). Mixtures of increasing polarity of heptane and ethyl acetate (EtOAc), dicloromethane (DCM) and methanol (MeOH) or water and acetonitrile (ACN) with 0.1% of formic acid (H_2O (95:5:0.1) and ACN (95:5:0.1)) were used as eluents. ^1H NMR and ^{13}C spectra were recorded on a Varian AS400 (400 MHz) spectrometer or on a Bruker Avance (400 MHz) spectrometer. Chemical shifts are reported as δ downfield in parts per million (ppm) using the residual solvent signal as internal reference. Coupling constants (*J* values) are given in hertz (Hz) and multiplicities are reported using the following abbreviation (s = singlet, d = doublet, t = triplet, q = quartet, m = multiplet, bs = broad signal).

Synthesis

General: All assayed compounds were characterized by ^1H NMR, ^{13}C NMR, HPLC-MS and HRMS and had a purity ≥95% by UPLC-MS analysis.

Scheme 1: Synthesis of macrocycles 5a and 5b.[a]

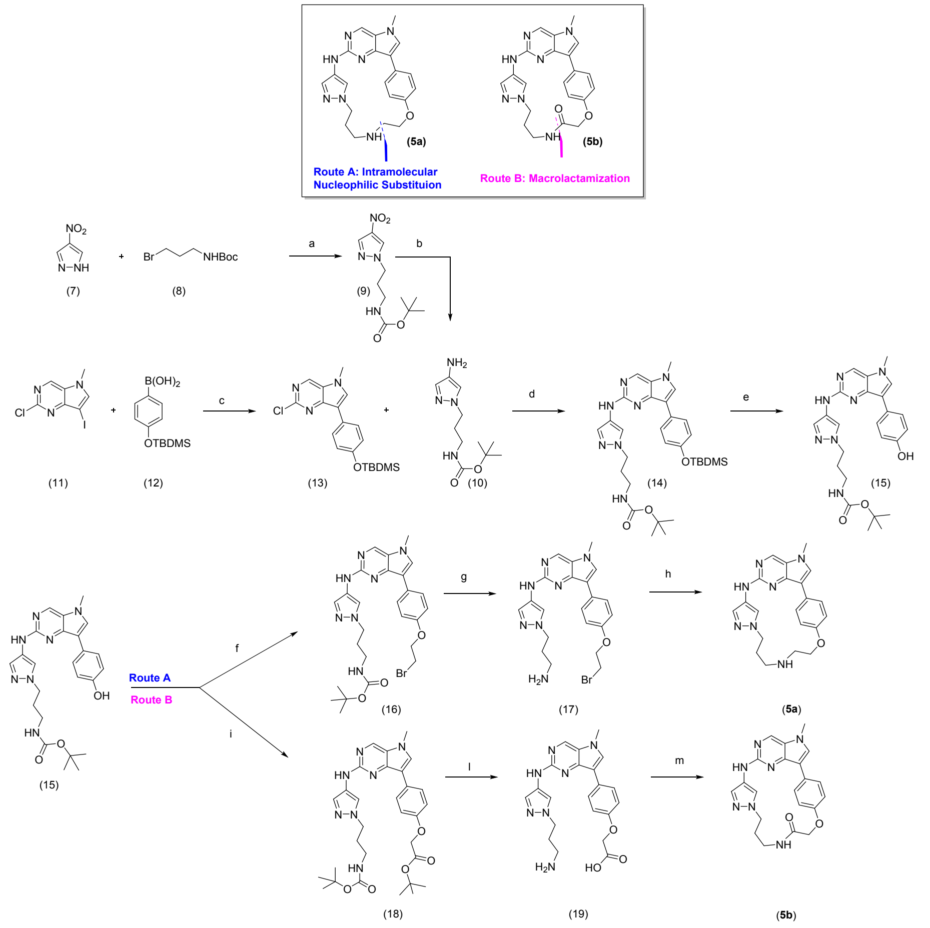

[a]**Reagents and conditions**: (a) K_2CO_3, dry ACN, 60°C, 4h ,99%; (b) H_2, Pd/C, EtOH, r.t., quantitative; (c) K_3PO_4, XPhos Pd(crotyl)Cl, Dioxane, 60°C, 24 h, 65%; (d) Cs_2CO_3, RuPhos Pd(crotyl)Cl, Dioxane, 80°C, 4h, 80%; (e) TBAF, THF, r.t., 2 h, quantitative; (f) 1,2-Dibromoethane, K_2CO_3, dry ACN, 60°C, overnight, 45%; (g) HBr 47%, ACN, r.t., 1 h, quantitative; (h) Et_3N, Dioxane/2-Methoxyethanol 10:2, 100°C, 72 h; 10% (i) *tert*-Butyl bromoacetate, K_2CO_3, dry ACN, 70°C, overnight, 50%; (l) 4N HCl, dioxane, r.t., 2 h, quantitative on crude; (m) HATU, Et_3N, dry DMF, r.t., overnight, 3.6%.

Scheme 1-Synthetic procedures:

Tert-butyl (3-(4-nitro-1H-pyrazol-1-yl)propyl)carbamate (9).

4-nitro-1H-pyrazole (0.5 g, 4.42 mmol), tert-butyl (3-bromopropyl)carbamate (1.16 g, 4.87 mmol) and K_2CO_3 (1.22 g, 8.82 mmol) were dissolved in dry ACN (7 mL). The reaction mixture was stirred at 60°C for 4 hours. Afterward, ACN was evaporated under reduced pressure, the residue was washed with water (2x 100 mL) and extracted with EtOAc (200 mL). The organic layer was dried with Na_2SO_4,

filtered, and evaporated under reduced pressure. The pure product was obtained in 99% yield and directly used with no further purification.

^{1}H NMR (400 MHz, $CDCl_3$) δ (ppm)= 8.26 (s, 1H), 8.09 (s, 1H), 4.72 (s, 1H), 4.24 (t, *J* = 6.7 Hz, 2H), 3.18 (t, *J* = 6.1 Hz, 2H), 2.10 (p, *J* = 6.6 Hz, 2H), 1.47 (s, 9H).

UPLC-MS (Method 1): t_R= 0.88 min; MS (ESI): m/z 215.2 $[M\text{-}tBu]^+$; 171.3 $[M\text{-}Boc]^+$

Tert-butyl (3-(4-amino-1H-pyrazol-1-yl)propyl)carbamate (10).

To a stirred solution of tert-butyl (3-(4-nitro-1H-pyrazol-1-yl)propyl)carbamate (0.45 g, 1.66 mmol) in EtOH (5 mL), palladium on carbon (0.018 g, 0.169 mmol) was added under N_2 atmosphere. The resulting solution was stirred for 1 day at room temperature under hydrogen pressure. The reaction solution was filtered through a Celite pad to remove the Pd/C catalyst and concentrated to obtain the target compound in quantitative yield without further purification.

^{1}H NMR (400 MHz, $CDCl_3$) δ (ppm)= 7.22 (s, 1H), 7.16 (s, 1H), 4.84 (t, *J* = 6.3 Hz, 1H), 4.09 (t, *J* = 6.6 Hz, 2H), 3.76 (bs, 2H), 3.09 (q, *J* = 6.5 Hz, 2H), 1.98 (p, *J* = 6.6 Hz, 2H), 1.46 (s, 9H).

UPLC-MS (Method 1): t_R= 0.33 min; MS (ESI): m/z 241.2 $[M+H]^+$

7-(4-((tert-butyldimethylsilyl)oxy)phenyl)-2-chloro-5-methyl-5H-pyrrolo[3,2-d]pyrimidine (12).

In a dry two neck bottomed flask, 2-chloro-7-iodo-5-methyl-5H-pyrrolo[3,2-d]pyrimidine (**13**) (0.1 g, 0.34 mmol), (4-((tert-butyldimethylsilyl)oxy)phenyl)boronic acid (**12**) (0.128 g, 0.51 mmol), and K_3PO_4 (0.651 g, 1.02 mmol) were dissolved in about 5 mL of dry dioxane. N_2 was fluxed into the solution for 5 minutes, and then XPhos Pd(crotyl)Cl (0.014 g, 0.02 mmol) was added. The reaction mixture was stirred at 60°C for 1 day. Afterward, dioxane was evaporated under reduced pressure, the residue was washed with water (2x50 mL) and extracted with EtOAc (100 mL). The organic layer was dried with Na_2SO_4, filtered, and evaporated to dryness under reduced pressure. The crude product was purified through flash chromatography on silica gel (DCM/EtOAc 95:5) to obtain the pure product in 65% yield.

^{1}H NMR (400 MHz, $CDCl_3$) δ (ppm)= 8.67 (s, 1H), 7.88 (d, *J* = 8 Hz, 2H), 7.64 (s, 1H), 6.94 (d, *J* = 8 Hz, 2H), 3.93 (s, 3H), 1.02 (s, 9H), 0.24 (s, 6H).

UPLC-MS (Method 1): t_R= 1.53 min; MS (ESI): m/z 374.28 $[M+H]^+$

Tert-butyl (3-(4-((7-(4-((tert-butyldimethylsilyl)oxy)phenyl)-5-methyl-5H-pyrrolo[3,2-d]pyrimidin-2-yl)amino)-1H-pyrazol-1-yl)propyl)carbamate (14)

In a dry two neck bottomed flask, 7-(4-((tert-butyldimethylsilyl)oxy)phenyl)-2-chloro-5-methyl-5H-pyrrolo[3,2-d]pyrimidine (**13**) (0.023 g, 0.06 mmol), tert-butyl (3-(4-amino-1H-pyrazol-1-yl)propyl)carbamate (**10**) (0.017 g, 0.07 mmol), and Cs_2CO_3 (0.06 g, 0.18 mmol) were dissolved in

about 1 mL of dry dioxane. N_2 was fluxed into the solution for 5 minutes, and then RuPhos Pd(crotyl)Cl (0.008 g, 0.012 mmol) was added. The reaction mixture was stirred at 80°C for 4 hours. Afterward, dioxane was evaporated under reduced pressure, the residue was washed with water (2x25 mL) and extracted with EtOAc (50 mL). The organic layer was dried with Na_2SO_4, filtered, and evaporated to dryness under reduced pressure. The crude product was purified through flash chromatography on silica gel (EtOAc 100%) to obtain the pure product in 80% yield.

^{1}H NMR (400 MHz, $CDCl_3$) δ (ppm)= 8.51 (s, 1H), 8.09 (s, 1H), 7.89 (d, *J* = 8.6 Hz, 2H), 7.59 (s, 1H), 7.47 (s, 1H), 7.13 (bs, 1H), 6.93 (d, *J* = 8.6 Hz, 2H), 4.90 (bs, 1H), 4.20 (t, *J* = 6.6 Hz, 2H), 3.85 (s, 3H), 3.16 – 3.11 (m, 2H), 2.10 – 2.024(m, 4H), 1.44 (s, 9H), 1.02 (s, 9H), 0.25 (s, 6H).

UPLC-MS (Method 1): t_R= 1.38 min; MS (ESI): m/z 578.5 $[M+H]^+$

Tert-butyl (3-(4-((7-(4-hydroxyphenyl)-5-methyl-5H-pyrrolo[3,2-d]pyrimidin-2-yl)amino)-1H-pyrazol-1-yl)propyl)carbamate (15).

To a stirred solution of tert-butyl (3-(4-((7-(4-((tert-butyldimethylsilyl)oxy)phenyl)-5-methyl-5H-pyrrolo[3,2-d]pyrimidin-2-yl)amino)-1H-pyrazol-1-yl)propyl)carbamate (**14**) (0.17 g, 0.29 mmol) in THF (2 mL), TBAF (0.1 g, 0.32 mmol) was added. The reaction was stirred at room temperature for 2 hours. Afterward, THF was evaporated under reduced pressure, the residue was washed with water (4x50 mL) and extracted with EtOAc (100 mL). The organic layer was dried with Na_2SO_4, filtered, and evaporated to dryness under reduced pressure. The desired product was recorded in quantitative yield and directly used with no further purification.

^{1}H NMR (400 MHz, CD_3OD) δ (ppm)= 8.59 (s, 1H), 8.17 (s, 1H), 7.92 (d, *J* = 8.6 Hz, 2H), 7.74 (s, 1H), 7.70 (s, 1H), 6.89 (d, *J* = 8.6 Hz, 2H), 4.18 (t, *J* = 6.9 Hz, 2H), 3.89 (s, 3H), 3.11 (t, *J* = 6.7 Hz, 2H), 2.07 (p, *J* = 6.9 Hz, 2H), 1.43 (s, 9H).

UPLC-MS (Method 1): t_R= 0.75 min; MS (ESI): m/z 464.4 $[M+H]^+$

Route A-Synthetic Procedure

Tert-butyl (3-(4-((7-(4-(2-bromoethoxy)phenyl)-5-methyl-5H-pyrrolo[3,2-d]pyrimidin-2-yl)amino)-1H-pyrazol-1-yl)propyl)carbamate (16).

Tert-butyl (3-(4-((7-(4-hydroxyphenyl)-5-methyl-5H-pyrrolo[3,2-d]pyrimidin-2-yl)amino)-1H-pyrazol-1-yl)propyl)carbamate (**15**) (0.135 g, 0.29 mmol) was dissolved in dry ACN (2 ml) and then K_2CO_3 (0.044 g, 0.32 mmol) and 1,2-dibromoethane (0.15 mL, 1.75 mmol) were added. The reaction mixture was stirred at 60°C overnight. Afterward, ACN was evaporated under reduced pressure, the residue was washed with water (2x50 mL) and extracted with EtOAc (100 mL). The organic layer was dried with Na_2SO_4, filtered, and evaporated to dryness under reduced pressure. The crude

product was purified through flash chromatography on silica gel (DCM/MeOH 95:5) to obtain the pure product in 45% yield.

^{1}H NMR (400 MHz, CD_2Cl_2) δ (ppm)= 8.55 (s, 1H), 8.10 (s, 1H), 8.02 (d, *J* = 8.8 Hz, 2H), 7.60 (s, 1H), 7.52 (s, 1H), 7.29 (s, 1H), 7.04 (d, *J* = 8.8 Hz, 2H), 4.39 (t, *J* = 6.0 Hz, 2H), 4.20 (t, *J* = 6.7 Hz, 2H), 3.84 (s, 3H), 3.73 (t, *J* = 6.0 Hz, 2H), 3.15 (q, *J* = 6.4 Hz, 2H), 2.08 (p, *J* = 6.7 Hz, 2H), 1.45 (s, 11H).

UPLC-MS (Method 1): t_R= 1.02; MS (ESI): m/z 570. 3 $[M+H]^+$

N-(1-(3-aminopropyl)-1H-pyrazol-4-yl)-7-(4-(2-bromoethoxy)phenyl)-5-methyl-5H-pyrrolo[3,2-d]pyrimidin-2-amine (17).

Tert-butyl (3-(4-((7-(4-(2-bromoethoxy)phenyl)-5-methyl-5H-pyrrolo[3,2-d]pyrimidin-2-yl)amino)-1H-pyrazol-1-yl)propyl)carbamate (**16**) (0.065 g, 0.11 mmol) was dissolved in ACN (4 mL) and then 47% aqueous HBr (20 µL, 0.14 mmol) was added. The solution was stirred at room temperature for 1 hour. After reaction completion the solvent was removed under reduced pressure to give the desired product in quantitative yield which was used without further purification.

UPLC-MS (Method 1): t_R= 0.58; MS (ESI): m/z 470.3 $[M+H]^+$

(E)-25-methyl-25H,41H-11-oxa-3,8-diaza-2(7,2)-pyrrolo[3,2-d]pyrimidina-4(4,1)-pyrazola-1(1,4)-benzenacycloundecaphane (5a)

N-(1-(3-aminopropyl)-1H-pyrazol-4-yl)-7-(4-(2-bromoethoxy)phenyl)-5-methyl-5H-pyrrolo[3,2-d]pyrimidin-2-amine (**14**) (0.053 g, 0.11 mmol) was dissolved in a 10:2 dioxane/2-methoxyethanol mixture (12 mL). Afterward, TEA (0.07 mL, 1.35 mmol) was added, and the reaction mixture was stirred at 100°C for 3 days. After reaction completion the solvent was removed under reduced pressure and the residue was purified through reverse phase HPLC (Method 1) to obtain the pure product (formate salt) in a 10% yield.

^{1}H NMR (400 MHz, CD_3OD) δ (ppm)= 8.63 (s, 1H), 8.62 (s, 1H), 8.57 (s, 2H), 7.73 (d, *J* = 8.7 Hz, 2H), 7.67 (s, 1H), 7.34 (d, *J* = 0.7 Hz, 1H), 7.24 (d, *J* = 8.7 Hz, 2H), 4.41 (t, *J* = 5.9 Hz, 2H), 4.18 – 4.13 (m, 2H), 3.93 (s, 3H), 2.85 – 2.77 (m, 4H), 2.01 – 1.95 (m, 2H).

13C NMR (151 MHz, CD_3OD) δ ppm 156.68 (1 C, s) 156.23 (1 C, s) 151.06 (1 C, s) 141.44 (1 C, s) 134.00 (1 C, s) 131.37 (1 C, s) 129.81 (1 C, s) 129.17 (1 C, s) 126.44 (1 C, s) 125.42 (1 C, s) 121.11 (1 C, s) 118.77 (1 C, s) 115.91 (1 C, s) 67.43 (1 C, s) 50.66 (1 C, s) 45.73 (1 C, s) 45.35 (1 C, s) 33.68 (1 C, s) 29.03 (1 C, s)

UPLC-MS (Method 4): t_R= 0.38; MS (ESI): m/z 390.3 $[M+H]^+$

UPLC Purity > 95%

Route B--Synthetic Procedure

Tert-butyl 2-(4-(2-((1-(3-((tert-butoxycarbonyl)amino)propyl)-1H-pyrazol-4-yl)amino)-5-methyl-5H-pyrrolo[3,2-d]pyrimidin-7-yl)phenoxy)acetate (18)

Tert-butyl (3-(4-((7-(4-hydroxyphenyl)-5-methyl-5H-pyrrolo[3,2-d]pyrimidin-2-yl)amino)-1H-pyrazol-1-yl)propyl)carbamate (**15**) (0.15 g, 0.32 mmol) was dissolved in dry ACN (4 mL) and then K_2CO_3 (0.05 g, 0.35 mmol) and *tert*-Butyl bromoacetate (0.052 mL, 0.35 mmol) were added. The reaction mixture was stirred at 70°C overnight. Afterward, ACN was evaporated under reduced pressure, the residue was washed with water (2x50 mL) and extracted with EtOAc (100 mL). The organic layer was dried with Na_2SO_4, filtered, and evaporated to dryness under reduced pressure. The crude product was purified through flash chromatography on silica gel (DCM/MeOH 95:5) to obtain the pure product in 50% yield.

^{1}H NMR (400 MHz, CD_2Cl_2) δ (ppm)= 8.56 (s, 1H), 8.11 (s, 1H), 8.02 (d, *J* = 8.8 Hz, 2H), 7.58 (s, 1H), 7.53 (s, 1H), 7.19 (s, 1H), 7.01 (d, *J* = 8.8 Hz, 2H), 5.01 (t, *J* = 6.2 Hz, 1H), 4.60 (s, 2H), 4.20 (t, *J* = 6.7 Hz, 2H), 3.85 (s, 3H), 3.14 (q, *J* = 6.5 Hz, 2H), 2.13 – 2.01 (m, 2H), 1.54 (s, 9H), 1.45 (s, 10H).

UPLC-MS (Method 1): t_R= 1.07; MS (ESI): m/z 578.4 $[M+H]^+$

2-(4-(2-((1-(3-aminopropyl)-1H-pyrazol-4-yl)amino)-5-methyl-5H-pyrrolo[3,2-d]pyrimidin-7-yl)phenoxy)acetic acid (19)

Tert-butyl 2-(4-(2-((1-(3-((tert-butoxycarbonyl)amino)propyl)-1H-pyrazol-4-yl)amino)-5-methyl-5H-pyrrolo[3,2-d]pyrimidin-7-yl)phenoxy)acetate (**18**) (0.1 g, 0.17 mmlol) was dissolved in 2 mL of 4M HCl solution in dioxane. The reaction was stirred at room temperature for 2 hours. After reaction completion the solvent was removed under reduced pressure to give the desired product in quantitative yield which was used without further purification.

UPLC-MS (Method 1): t_R= 0.42; MS (ESI): m/z 422.3 [M+H]+

(E)-25-methyl-25H,41H-11-oxa-3,8-diaza-2(7,2)-pyrrolo[3,2-d]pyrimidina-4(4,1)-pyrazola-1(1,4)-benzenacycloundecaphan-9-one (5b)

2-(4-(2-((1-(3-aminopropyl)-1H-pyrazol-4-yl)amino)-5-methyl-5H-pyrrolo[3,2-d]pyrimidin-7-yl)phenoxy)acetic acid (**19**) (0.075 g, 0.18 mmol) was dissolved in 7 mL of dry DMF. Afterward, HATU (0.08 g, 0.21 mmol) was added. The reaction mixture was stirred at room temperature for 30 minutes and then TEA (0.08 mL, 0.54 mmol) was added. The reaction was stirred at room temperature for 1 hour. After reaction completion the solvent was removed under reduced pressure. The crude product was purified through reverse phase flash chromatography (Biotage Isolera, Sfar C18 cartridge,

gradient of elution of 100:0 A to 60:40 A/B in 12 CV; A: water/ACN 95:5 + 0.1% HCOOH, B: ACN/water 95:5 + 0.1% HCOOH). Appropriate fractions were combined and evaporated under reduced pressure to give (E)-25-methyl-25H,41H-11-oxa-3,8-diaza-2(7,2)-pyrrolo[3,2-d]pyrimidina-4(4,1)-pyrazola-1(1,4)-benzenacycloundecaphan-9-one, 2Formic Acid (3.2 mg, 6.46 µmol, 3.63 % yield)

^{1}H NMR (DMSO-d6, 400 MHz) δ (ppm)= 9.16 (s, 1H), 8.74 (br s, 1H), 8.70 (s, 1H), 7.83 (s, 1H), 7.77 (d, 2H, J=8.8 Hz), 7.19 (s, 1H), 7.02 (d, 2H, J=8.6 Hz), 4.63 (s, 2H), 4.1-4.1 (m, 2H), 3.83 (s, 3H), 3.1-3.2 (m, 2H), 1.75 (br dd, 2H, J=3.7, 5.5 Hz)

^{13}C NMR (101 MHz, DMSO-d6) δ (ppm)= 168.08 (1 C, s), 155.67 (1 C, s), 154.54 (1 C, s), 147.83 (1 C, s), 141.33 (1 C, s), 131.69 (1 C, s), 128.40 (2 C, s), 127.80 (1 C, s), 126.13 (1 C, s), 124.69 (1 C, s), 123.45 (1 C, s), 118.48 (1 C, s), 114.06 (2 C, s), 112.48 (1 C, s), 66.39 (1 C, s), 49.77 (1 C, s), 35.61 (1 C, s), 33.16 (1 C, s), 31.84 (1 C, s)

Method 1, t_R: 0.55 min, MS (ESI): m/z 404.34 [M+H]$^+$

HPLC purity: >95%

Scheme 2-Synthetic procedures:

6a and **6b**. **Scheme 2: Synthesis of macrocycles 6a and 6b.**[a]

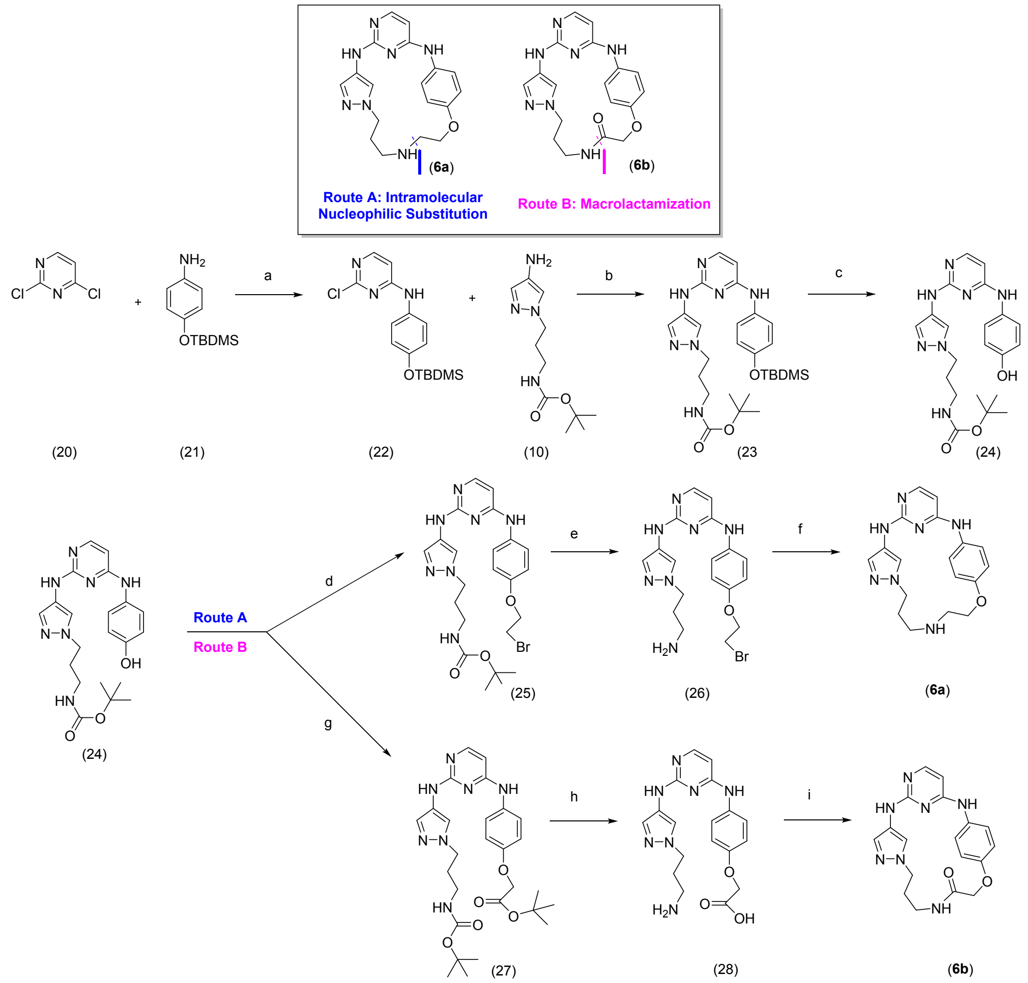

[a]**Reagents and conditions:** (a) Et_3N, EtOH, r.t, overnight, 92%; (b) K_2CO_3, dioxane, 80°C, overnight, 70%; (c) TBAF, THF, r.t., 2 h, quantitative; (d) 1,2-Dibromoethane, K_2CO_3, dry ACN, 60°C, overnight, 30%; (e) HBr 47%, ACN, r.t., 1 hr, quantitative on crude; (f) Et_3N, Dioxane/2-Methoxyethanol 10:2, 100°C, 1 day, 20%; (g) *tert*-Butyl bromoacetate, K_2CO_3, dry ACN, 70°C, overnight, 55%; (h) 4N HCl, dioxane, r.t., 2 h, quantitative on crude; (i) HATU, Et_3N, dry DMF, r.t., 2 h, 10%.

N-(4-((tert-butyldimethylsilyl)oxy)phenyl)-2-chloropyrimidin-4-amine (22)

To a stirred solution of 2,4-dichloropyrimidine (0.1 g, 0.67 mmol) in absolute EtOH (5 mL), TEA (0.14 mL, 1 mmol) and 4-((tert-butyldimethylsilyl)oxy)aniline (0.15 g, 0.67 mmol) were added. The reaction mixture was stirred at room temperature overnight. Afterward, EtOH was evaporated under reduced pressure. The pure product was obtained after recrystallization from cold ethanol in 92% yield.

^1H NMR (400 MHz, $CDCl_3$) δ (ppm)= 8.09 (d, J = 5.9 Hz, 1H), 7.16 (d, 2H), 7.00 (s, 1H), 6.90 (d, 2H), 6.47 (d, J = 6.0 Hz, 1H), 1.02 (s, 9H), 0.24 (s, 6H).

UPLC-MS (Method 1): t_R= 1.43 min; MS (ESI): m/z 336.2 $[M+H]^+$

Tert-butyl (3-(4-((4-((4-((tert-butyldimethylsilyl)oxy)phenyl)amino)pyrimidin-2-yl)amino)-1H-pyrazol-1-yl)propyl)carbamate (23)

N-(4-((tert-butyldimethylsilyl)oxy)phenyl)-2-chloropyrimidin-4-amine (**22**) (0.2 g, 0.59 mmol) was dissolved in 2.5 mL of dioxane, and then K_2CO_3 (0.09 g, 0.65 mmol) and tert-butyl(3-(4-amino-lH-pyrazo-l-yl)propyl)carbamate (**10**) (0.144 g, 0.59 mmol) were added. The reaction mixture was stirred at reflux overnight. Afterward, dioxane was evaporated under reduced pressure, the residue was washed with water (2x50 mL) and extracted with EtOAc (100 mL). The organic layer was dried with Na_2SO_4, filtered, and evaporated to dryness under reduced pressure. The crude product was purified through flash chromatography on silica gel (EtOAc 100%) to obtain the pure product in 70% yield.

^{1}H NMR (400 MHz, $CDCl_3$) δ (ppm)= 7.98 (d, *J* = 5.8 Hz, 1H), 7.94 (s, 1H), 7.44 (s, 1H), 7.19 (d, *J* = 8.7 Hz, 2H), 6.86 (s, 2H), 6.77 (bs, 1H), 6.71 (bs, 1H), 6.00 (d, *J* = 5.8 Hz, 1H), 4.79 (s, 1H), 4.16 (t, *J* = 6.5 Hz, 2H), 3.26 – 3.13 (m, 2H), 2.01 (q, *J* = 6.5 Hz, 2H), 1.43 (s, 9H), 1.02 (s, 9H), 0.24 (s, 6H).

UPLC-MS (Method 1): t_R= 0.98 min; MS (ESI): m/z 540.4 $[M+H]^+$

Tert-butyl (3-(4-((4-((4-hydroxyphenyl)amino)pyrimidin-2-yl)amino)-1H-pyrazol-1-yl)propyl)carbamate (24).

To a stirred solution of tert-butyl (3-(4-((4-((4-((tert-butyldimethylsilyl)oxy) phenyl)amino)pyrimidin-2-yl)amino)-1H-pyrazol-1-yl)propyl)carbamate (**23**) (0.1 g, 0.18 mmol) in THF (2 mL), TBAF (0.064 g, 0.20 mmol) was added. The reaction was stirred at room temperature for 2 hours. Afterward, THF was dried at rotavapor, the residue was washed with water (4x25 mL) and extracted with EtOAc (50 mL). The organic layer was dried with Na_2SO_4, filtered, and evaporated to dryness under reduced pressure. The desired product was recorded in quantitative yield and directly used with no further purification.

^{1}H NMR (400 MHz, DMSO-d6) δ (ppm)= 9.21 (bs, 1H), 8.90 (d, *J* = 15.1 Hz, 2H), 7.87 (d, *J* = 5.8 Hz, 1H), 7.79 (bs, 1H), 7.41 (s, 1H), 7.32 (bs, 1H), 6.87 (d, *J* = 6.0 Hz, 1H), 6.80 – 6.70 (m, 2H), 5.98 (d, *J* = 5.6 Hz, 1H), 3.98 (t, 2H), 2.90 (q, *J* = 6.5 Hz, 2H), 1.82 (p, *J* = 7.0 Hz, 2H), 1.38 (s, 9H).

UPLC-MS (Method 1): t_R= 0.54 min; MS (ESI): m/z 426.4 $[M+H]^+$

Tert-butyl (3-(4-((4-((4-(2-bromoethoxy)phenyl)amino)pyrimidin-2-yl)amino)-1H-pyrazol-1-yl)propyl)carbamate (25).

Intermediate **24** (0.135 g, 0.29 mmol) was dissolved in dry ACN (2 mL) and then K_2CO_3 (0.044 g, 0.32 mmol) and 1,2-dibromoethane (0.15 mL, 1.75 mmol) were added. The reaction mixture was stirred at 60°C overnight. Afterward, ACN was evaporated under reduced pressure, the residue was

washed with water (2x50 mL) and extracted with EtOAc (100 mL). The organic layer was dried with Na_2SO_4, filtered, and evaporated to dryness under reduced pressure. The crude product was purified through flash chromatography on silica gel (DCM/MeOH 95:5) to obtain the pure product in 30% yield.

^{1}H NMR (400 MHz, CD_3OD) δ (ppm)= 7.85 (d, *J* = 6.0 Hz, 1H), 7.81 (s, 1H), 7.49 (s, 1H), 7.43 (d, *J* = 9.0 Hz 2H), 7.00 (d, *J* = 9.0 Hz, 2H), 6.07 (d, *J* = 6.0 Hz, 1H), 4.35 (d, *J* = 6.3, 5.1 Hz, 2H), 4.07 (t, *J* = 6.8 Hz, 2H), 3.74 (d, *J* = 6.2, 5.2 Hz, 2H), 3.04 (t, *J* = 6.8 Hz, 2H), 2.04 – 1.88 (m, 2H), 1.44 (s, 9H).

UPLC-MS (Method 1): t_R= 0.72 min; MS (ESI): m/z 532.2-534.2 $[M+H]^+$

N2-(1-(3-aminopropyl)-1H-pyrazol-4-yl)-N4-(4-(2-bromoethoxy)phenyl)pyrimidine-2,4-diamine (26).

Tert-butyl (3-(4-((4-((4-(2-bromoethoxy)phenyl)amino)pyrimidin-2-yl)amino)-1H-pyrazol-1-yl)propyl)carbamate (**25**) (0.14 g, 0.26 mmol) was dissolved in ACN (5 ml) and then 47% aqueous HBr (46 µL, 0.312 mmol) was added. The solution was stirred at room temperature for 1 hour. After reaction completion the solvent was removed under reduced pressure to give the desired product, in quantitative yield, which was used without further purification.

UPLC-MS (Method 1): t_R= 0.36 min; MS (ESI): m/z 432.2-434.2 $[M+H]^+$

(E)-11H-6-oxa-2,4,9-triaza-3(2,4)-pyrimidina-1(4,1)-pyrazola-5(1,4)-benzenacyclododecaphane (6a).

N2-(1-(3-aminopropyl)-1H-pyrazol-4-yl)-N4-(4-(2-bromoethoxy)phenyl)pyrimidine-2,4-diamine (**26**) (0.12 g, 0.27 mmol) was dissolved in a 10:2 dioxane/2-methoxyethanol mixture (24 mL). Afterward, TEA (0.2 mL, 1.35 mmol) was added, and the reaction mixture was stirred at 100°C for 1 day. After reaction completion the solvent was removed under reduced pressure and the residue was triturated using a DCM/MeOH mixture. The solid was washed with water and centrifugated. The supernatant was evaporated under reduced pressure and purified using a cation exchange cartridge (SCX) eluting with 7N ammonia solution in MeOH to obtain the pure product in 20% yield.

^{1}H NMR (400 MHz, DMSO-d6) δ (ppm)= 8.90 (s, 1H), 7.82 (d, *J* = 5.6 Hz, 1H), 7.37 (d, *J* = 4.5 Hz, 1H), 7.18 (dd, *J* = 8.8, 1.7 Hz, 2H), 7.11 – 7.05 (m, 3H), 5.94 (d, *J* = 5.7 Hz, 1H), 4.29 – 4.20 (m, 2H), 3.88 – 3.80 (m, 2H), 2.67 (s, 2H), 2.57 (t, *J* = 5.7 Hz, 2H), 1.61 – 1.52 (m, 2H).

^{13}C NMR (400 MHz, DMSO-d6) δ (ppm)= 167.01, 162.98, 159.25, 156.56, 154.52, 132.76, 128.85, 127.91, 123.96, 119.36, 116.58, 96.25, 70.25, 55.29, 49.53, 44.55, 44.04, 7.79.

UPLC-MS (Method 3): t_R= 0.32 min; MS (ESI): m/z 352.3 $[M+H]^+$

UPLC Purity > 95%

Route B-synthetic procedure

Tert-butyl 2-(4-((2-((1-(3-((tert-butoxycarbonyl)amino)propyl)-1H-pyrazol-4 yl)amino)pyrimidin-4-yl)amino)phenoxy)acetate (27).

Tert-butyl(3-(4-((4-((4-hydroxyphenyl)amino)pyrimidin-2-yl)amino)-1H-pyrazol-1-yl)propyl) carbamate (**24**) (0.05 g, 0.12 mmol) was dissolved in 1 mL of dry ACN. Afterward, K_2CO_3 (0.02 g, 0.13 mmol) and *tert*-Butyl bromoacetate (0.02 mL, 0.13 mmol) were added and the reaction mixture was stirred at 70°C for 4 hours. Afterward, ACN was evaporated under reduced pressure, the residue was washed with water (2x25 mL) and extracted with EtOAc (50 mL). The organic layer was dried with Na_2SO_4, filtered, and evaporated to dryness under reduced pressure. The residue was purified through flash chromatography on silica gel (DCM/MeOH 95:5) to obtain the pure product in a 55% yield.

^{1}H NMR (400 MHz, CD_3OD) δ (ppm)= 7.87 – 7.78 (m, 2H), 7.49 (s, 1H), 7.43 (d, *J* = 8.5 Hz, 2H), 6.96 (d, *J* = 8.5 Hz, 2H), 6.07 (d, *J* = 6.0 Hz, 1H), 4.62 (s, 2H), 4.08 (t, *J* = 6.9 Hz, 2H), 3.05 (t, *J* = 6.7 Hz, 2H), 2.01 – 1.90 (m, 3H), 1.52 (s, 9H), 1.44 (s, 9H).

UPLC-MS (Method 1): t_R= 0.77; MS (ESI): m/z 540.4 $[M+H]^+$

2-(4-((2-((1-(3-aminopropyl)-1H-pyrazol-4-yl)amino)pyrimidin-4-yl)amino)phenoxy)acetic acid (28).

Tert-butyl 2-(4-((2-((1-(3-((tert-butoxycarbonyl)amino)propyl)-1H-pyrazol-4-yl)amino)pyrimidin-4-yl)amino)phenoxy)acetate (**27**) (0.035 g, 0.06 mmol) was dissolved in 1 mL of 4M HCl solution in dioxane. The reaction was stirred at room temperature for 1 hour. After reaction completion the solvent was removed under reduced pressure to give the desired product in quantitative yield which was used without further purification.

UPLC-MS (Method 1): t_R= 0.25; MS (ESI): m/z 384.3 $[M+H]^+$

(E)-11H-6-oxa-2,4,9-triaza-3(2,4)-pyrimidina-1(4,1)-pyrazola-5(1,4) benzenacyclododecaphan-8-one (6b).

2-(4-((2-((1-(3-aminopropyl)-1H-pyrazol-4-yl)amino)pyrimidin-4-yl)amino)phenoxy)acetic acid (**28**) (0.09 g, 0.23 mmol) was dissolved in 7 mL of dry DMF. Afterward, HATU (0.1 g, 0.28 mmol) was added. The reaction mixture was stirred at room temperature for 30 minutes and then TEA (0.11 mL, 0.7 mmol) was added. The reaction was stirred at room temperature for two hours. After reaction completion the solvent was removed under reduced pressure. The crude product was purified using a Biotage Isolera apparatus with prepacked silica gel column eluting with a DCM/ 7N NH_3 solution in MeOH mixture (Gradient: from 100% DCM to 20% DCM/7N NH_3 MeOH 8:2) to obtain the target compound in a 10% yield.

^{1}H NMR (400 MHz, DMSO-d6) δ (ppm)= 9.06 (s, 1H), 9.04 (s, 1H), 8.37 (t, *J* = 6.3 Hz, 1H), 7.89 (d, *J* = 5.6 Hz, 1H), 7.74 (s, 1H), 7.32 (d, *J* = 8.9 Hz, 2H), 7.14 (s, 1H), 6.99 (d, *J* = 8.9 Hz, 2H), 5.99 (d, *J* = 5.7 Hz, 1H), 4.58 (s, 2H), 3.87 – 3.76 (m, 2H), 3.21-3.16 (m, 2H), 1.70-1.64 (m, 2H).

^{13}C NMR (400 MHz, DMSO-d6) δ (ppm)= 168.95, 162.07, 159.31, 156.76, 154.57, 133.09, 128.90, 125.89, 125.83, 123.74, 119.87, 114.85, 96.54, 67.84, 49.74, 49.07, 35.17, 32.85.

UPLC-MS (Method 1): t_R= 0.33; MS (ESI): m/z 366.3 $[M+H]^+$

UPLC Purity > 95%

Analytical Methods

Analytical UPLC-MS analyses were carried out on a Waters ACQUITY UPLC equipped with a Photo Diode Array (PDA) detector and coupled with a Waters ACQUITY QDa single quadrupole mass spectrometer with an Electrospray ionization (ESI) source operating in alternating positive and negative ion mode.

HRMS analyses were performed using a Thermo Fisher Scientific Q Exactive mass spectrometer equipped with an HESI (Heated Electrospray Ionisation) ion source coupled with a Thermo Fisher Scientific Vanquish chromatographer. Samples were analyzed in positive ESI (ES+) ion mode, using a FIA (flow injection analysis) technique and processed with Excalibur (Thermo) software.

UPLC-MS Methods:

- **Method 1**: Phenomenex UPLC CSH C18 column (50mm x 2.1mm i.d. 1.7 µm particle size). Column Temperature (°C) 40.0. Mobile phases: 0.1% v/v solution of HCOOH in water (A); 0.1% v/v solution of HCOOH in ACN (B). Flow (mL/min) 1. Stop Time (mins) 2.0. Gradient:

**Time**	**Phase A**	**Phase B**
t_0: 0 min	99%	1%
t_1: 1.5 min	0.1%	99.9%
t_2: 1.9 min	0.1%	99.9%
t_3: 2 min	99%	1%

- **Method 2**: Kinetex UPLC CSH C8 column (50mm x 2.1mm i.d. 1.7 μm particle size). Column Temperature (°C) 40.0. Mobile phases: 0.1% v/v solution of HCOOH in water (A); 0.1% v/v solution of HCOOH in ACN (B). Flow (mL/min) 1. Stop Time (mins) 2.0. Gradient:

**Time**	**Phase A**	**Phase B**
t_0: 0 min	99%	1%
t_1: 1.5 min	0.1%	99.9%
t_2: 1.9 min	0.1%	99.9%
t_3: 2 min	99%	1%

- **Method 3**: Phenomenex UPLC CSH C18 column (50mm x 2.1mm i.d. 1.7 μm particle size). Column Temperature (°C) 40.0. Mobile phases: 0.1% v/v solution of HCOOH in water (A); 0.1% v/v solution of HCOOH in ACN (B). Flow (mL/min) 1. Stop Time (mins) 4.0. Gradient:

**Time**	**Phase A**	**Phase B**
t_0: 0 min	99%	1%
t_1: 3.5 min	0.1%	99.9%
t_2: 3.9 min	0.1%	99.9%
t_3: 4 min	99%	1%

- **Method 4**: Kinetex UPLC CSH C8 column (50mm x 2.1mm i.d. 1.7 μm particle size). Column Temperature (°C) 40.0. Mobile phases: 0.1% v/v solution of HCOOH in water (A); 0.1% v/v solution of HCOOH in ACN (B). Flow (mL/min) 1. Stop Time (mins) 4.0. Gradient:

**Time**	**Phase A**	**Phase B**

t_0: 0 min	99%	1%
t_1: 3.5 min	0.1%	99.9%
t_2: 3.9 min	0.1%	99.9%
t_3: 4 min	99%	1%

Preparative LC-MS Methods:

In some cases, compounds were purified by reverse phase HPLC using a Waters Fractionlynx preparative HPLC system (2525 pump, 2996/2998 UV/VIS detector, 2767 liquid handler) coupled to ACQUITY QDa Mass Detector. Fractions containing the required product (identified by MS analysis) were pooled and the solvent lyophilized. The purification was controlled by Waters Fractionlynx software through monitoring the set m/z. Appropriate separative methods were selected based on water and ACN solvent systems under acid conditions. The modifier used under acidic conditions was formic acid (0.1% V/V).

❖ **Method 1**: Kinetex HPLC C8 column (100mm x 21.2mm i.d. 5 µm particle size). Column Temperature (°C) 40.0. Mobile phases: water (A); ACN (B), 4% v/v solution of HCOOH in water (C). Flow (mL/min) 20. Gradient:

Time	Phase A	Phase B	Phase C
t_0: 0 min	95%	2.5%	2.5%
t_1: 1 min	95%	2.5%	2.5%
t_2: 12 min	2.5%	2.5%	2.5%
t_3: 14 min	95%	2.5%	2.5%
t_4: 14.10 min	95%	2.5%	2.5%

Summary of structural characterization and purity of macrocycles

No	^{1}H NMR	^{13}C NMR	HPLC purity/t_R	HRMS Exp. Mass Theor. Mass Proposed Molecular Formula $[M+H]^+$ Mass accuracy (ppm)
**5a**	(400 MHz, CD_3OD) δ (ppm) = 8.63 (s, 1H), 8.62 (s, 1H), 8.57 (s, 2H), 7.73 (d, *J* = 8.7 Hz, 2H), 7.67 (s, 1H), 7.34 (d, *J* = 0.7 Hz, 1H), 7.24 (d, *J* = 8.7 Hz, 2H), 4.41 (t, *J* = 5.9 Hz, 2H), 4.18 – 4.13 (m, 2H), 3.93 (s, 3H), 2.85 – 2.77 (m, 4H), 2.01 – 1.95 (m, 2H). Formate	(151 MHz, CD_3OD) δ (ppm) = 156.68 (1 C, s) 156.23 (1 C, s) 151.06 (1 C, s) 141.44 (1 C, s) 134.00 (1 C, s) 131.37 (1 C, s) 129.81 (1 C, s) 129.17 (1 C, s) 126.44 (1 C, s) 125.42 (1 C, s) 121.11 (1 C, s) 118.77 (1 C, s) 115.91 (1 C, s) 67.43	**Method 4** HPLC purity: >95%, t_R: 0.38 min	390.2034 390.2037 $C_{21}H_{24}ON_7$ -0.73

		(1 C, s) 50.66 (1 C, s) 45.73 (1 C, s) 45.35 (1 C, s) 33.68 (1 C, s) 29.03 (1 C, s)		
5b	(400 MHz, DMSO-d6,) δ (ppm) = 9.16 (s, 1H), 8.74 (br s, 1H), 8.70 (s, 1H), 7.83 (s, 1H), 7.77 (d, 2H, J=8.8 Hz), 7.19 (s, 1H), 7.02 (d, 2H, J=8.6 Hz), 4.63 (s, 2H), 4.1-4.1 (m, 2H), 3.83 (s, 3H), 3.1-3.2 (m, 2H), 1.75 (br dd, 2H, J=3.7, 5.5 Hz)	(101 MHz, DMSO-d6) δ (ppm) = 168.08 (1 C, s), 155.67 (1 C, s), 154.54 (1 C, s), 147.83 (1 C, s), 141.33 (1 C, s), 131.69 (1 C, s), 128.40 (2 C, s), 127.80 (1 C, s), 126.13 (1 C, s), 124.69 (1 C, s), 123.45 (1 C, s), 118.48 (1 C, s), 114.06 (2 C, s), 112.48 (1 C, s), 66.39 (1 C, s), 49.77 (1 C, s), 35.61 (1 C, s), 33.16 (1 C, s), 31.84 (1 C, s)	**Method 1** HPLC purity: >95%, t_R: 0.55 min	404.1826 404.1829 $C_{21}H_{22}O_2N_7$ -0.79

**6a**	(400 MHz, DMSO-d6) δ (ppm) = 8.90 (s, 1H), 7.82 (d, *J* = 5.6 Hz, 1H), 7.37 (d, *J* = 4.5 Hz, 1H), 7.18 (dd, *J* = 8.8, 1.7 Hz, 2H), 7.11 – 7.05 (m, 3H), 5.94 (d, *J* = 5.7 Hz, 1H), 4.29 – 4.20 (m, 2H), 3.88 – 3.80 (m, 2H), 2.67 (s, 2H), 2.57 (t, *J* = 5.7 Hz, 2H), 1.61 – 1.52 (m, 2H).	(400 MHz, DMSO-d6) δ (ppm) = 167.01, 162.98, 159.25, 156.56, 154.52, 132.76, 128.85, 127.91, 123.96, 119.36, 116.58, 96.25, 70.25, 55.29, 49.53, 44.55, 44.04, 7.79.	**Method 3** HPLC purity: >95%, t_R: 0.32 min.	352.1877 352.1880 $C_{18}H_{22}ON_7$ -0.87
**6b**	(400 MHz, DMSO-d6) δ (ppm) = 9.06 (s, 1H), 9.04 (s, 1H), 8.37 (t, *J* = 6.3 Hz, 1H), 7.89 (d, *J* = 5.6 Hz, 1H), 7.74 (s, 1H), 7.32 (d, *J* = 8.9 Hz, 2H), 7.14 (s, 1H), 6.99 (d, *J* = 8.9 Hz, 2H), 5.99 (d, *J* = 5.7 Hz, 1H), 4.58 (s, 2H), 3.87 – 3.76 (m,	(400 MHz, DMSO-d6) δ (ppm) = 168.95, 162.07, 159.31, 156.76, 154.57, 133.09, 128.90, 125.89, 125.83, 123.74, 119.87, 114.85, 96.54, 67.84, 49.74, 49.07, 35.17, 32.85.	**Method 1** HPLC purity: >95%, t_R: 0.33 min	366.1671 366.1673 $C_{18}H_{20}O_2N_7$ -0.52

	2H), 3.21-3.16 (m, 2H), 1.70-1.64 (m, 2H).			

NMR spectra

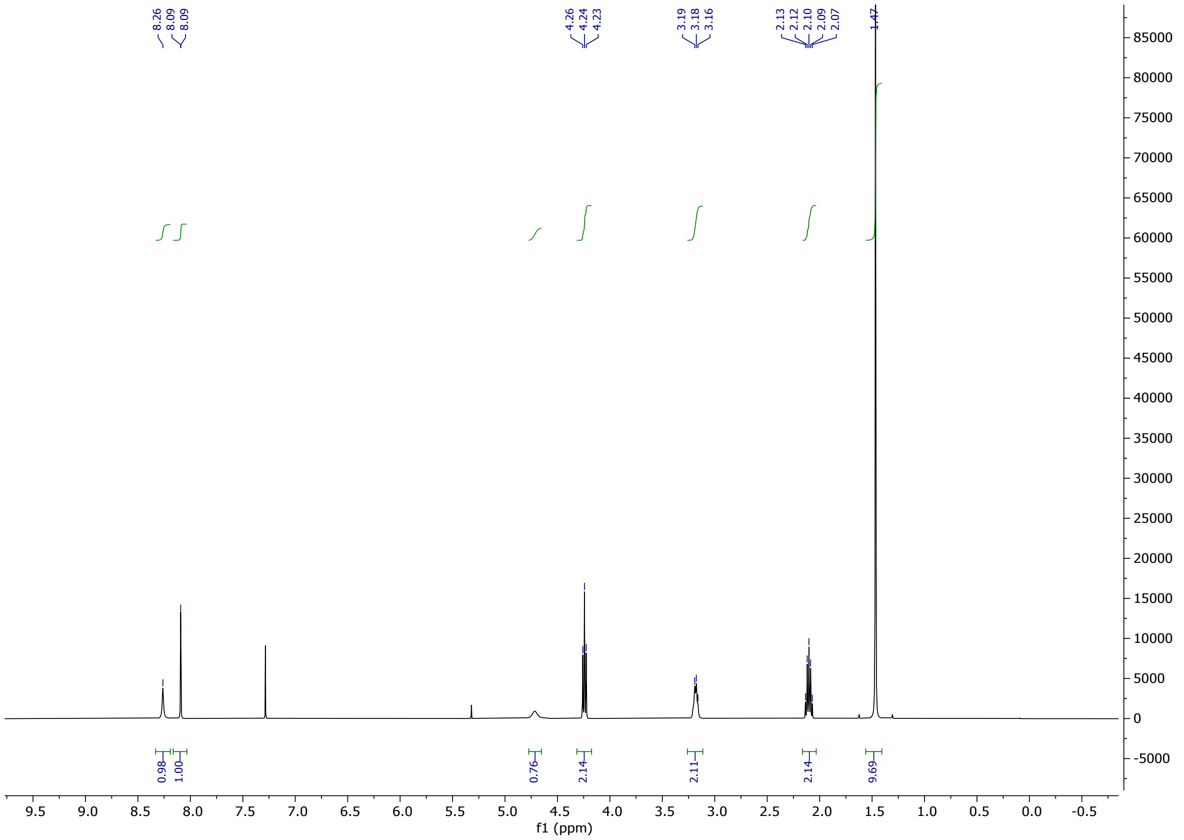

^{1}H spectrum ($CDCl_3$, 400 MHz, 298 K) of **intermediate 9**.

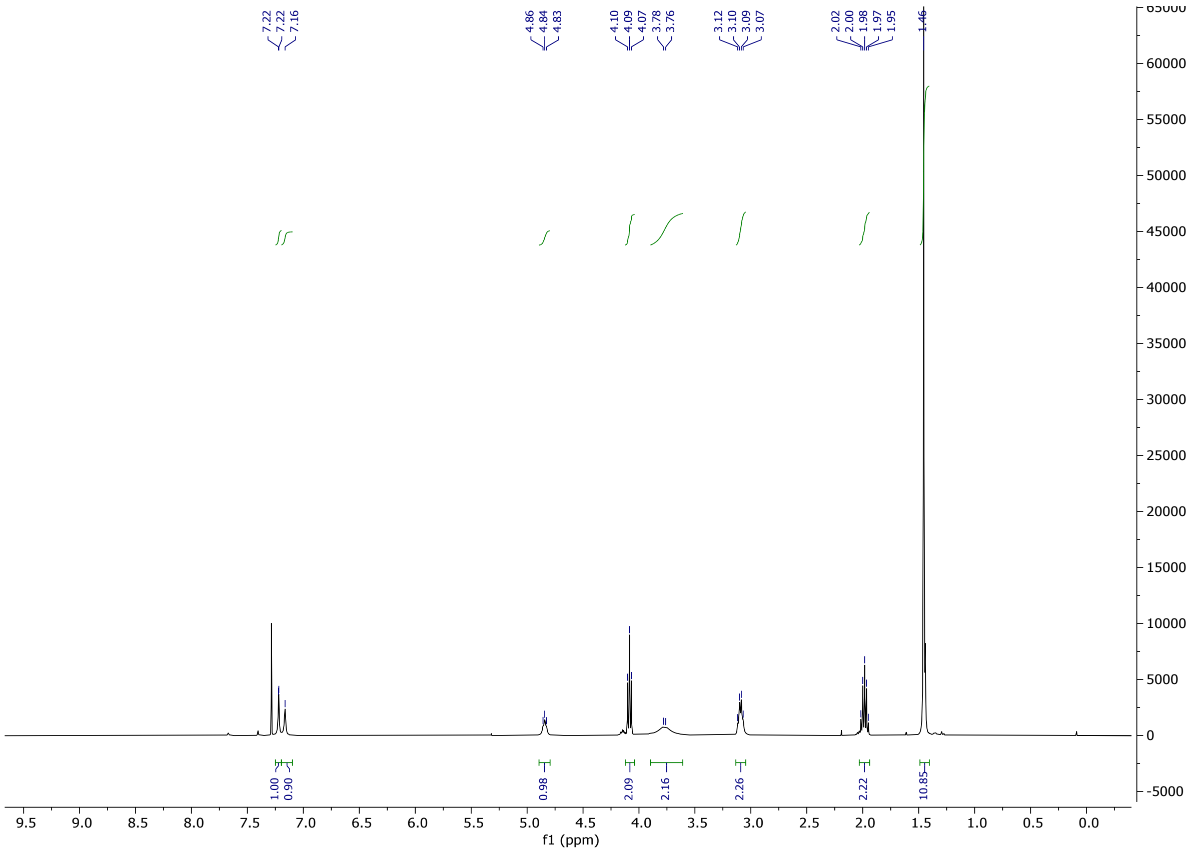

^1H spectrum ($CDCl_3$, 400 MHz, 298 K) of **intermediate 10.**

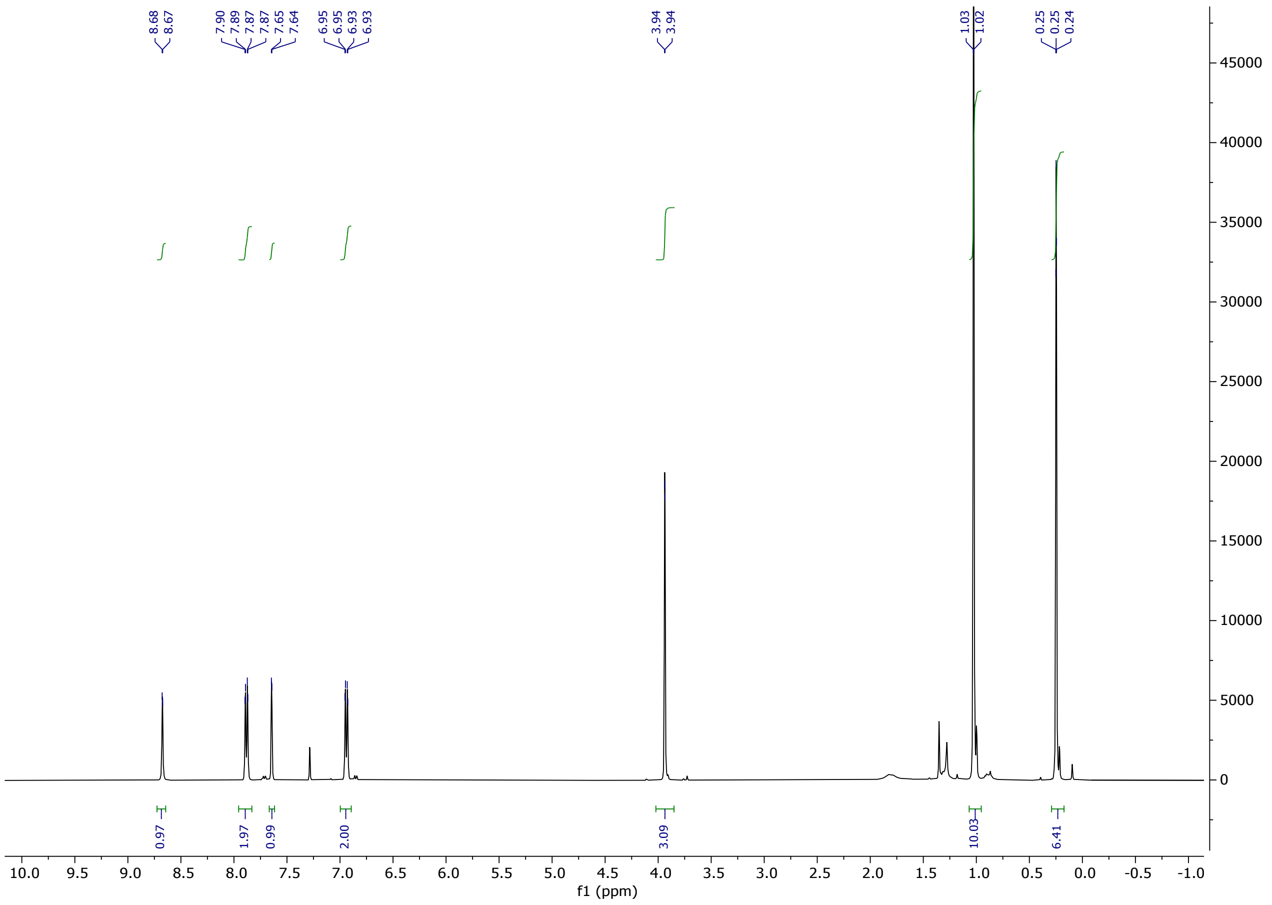

^1H spectrum ($CDCl_3$, 400 MHz, 298 K) of **intermediate 13.**

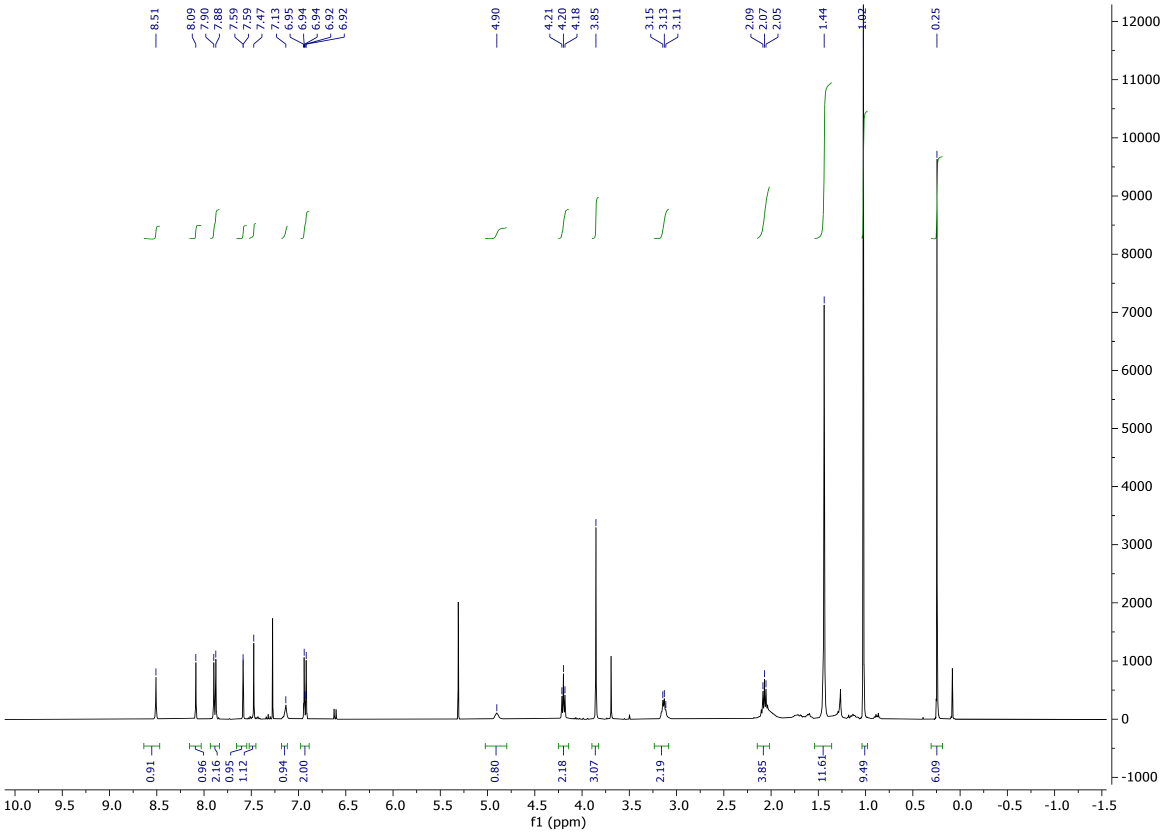

^{1}H spectrum ($CDCl_3$, 400 MHz, 298 K) of **intermediate 14.**

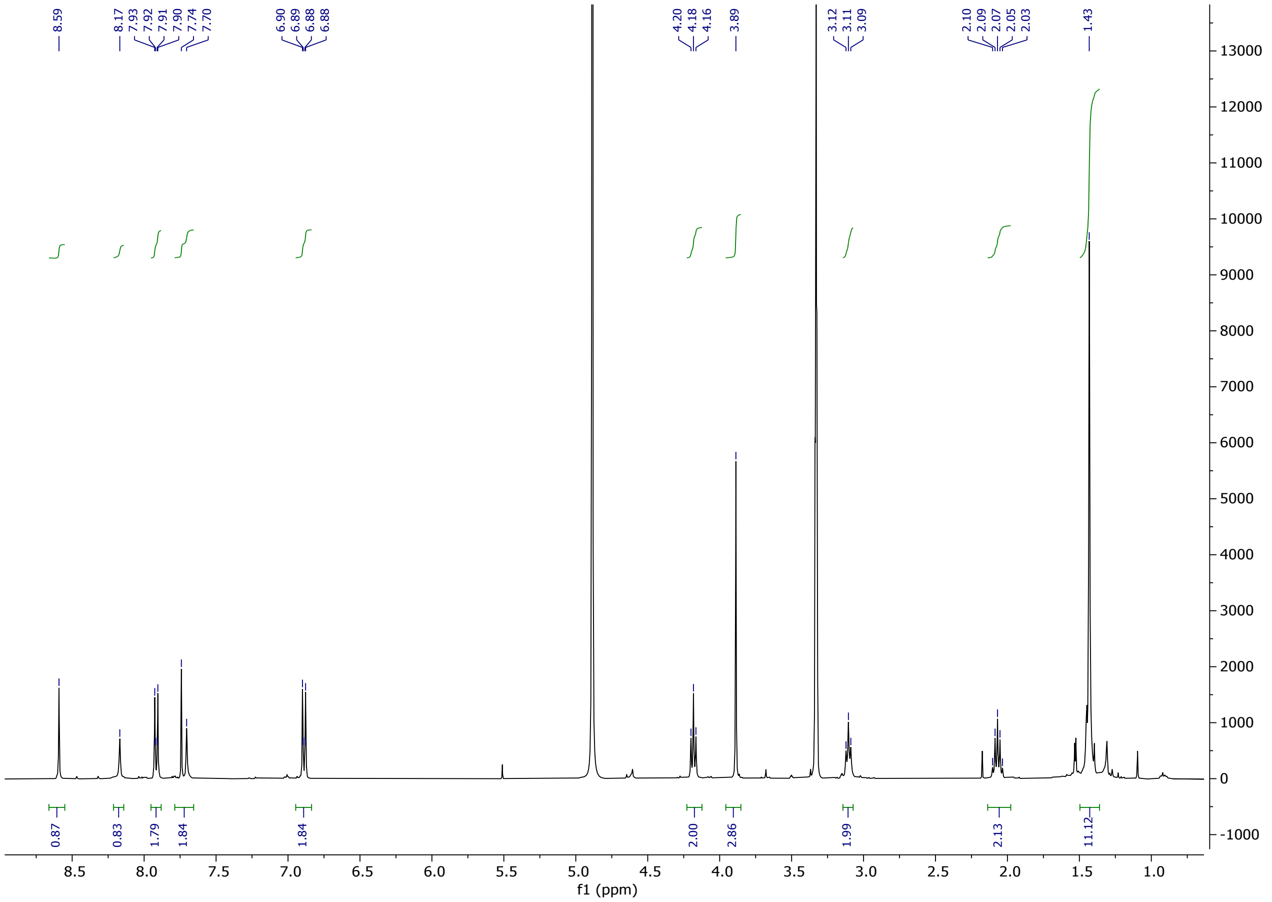

^{1}H spectrum (CD_3OD, 400 MHz, 298 K) of **intermediate 15.**

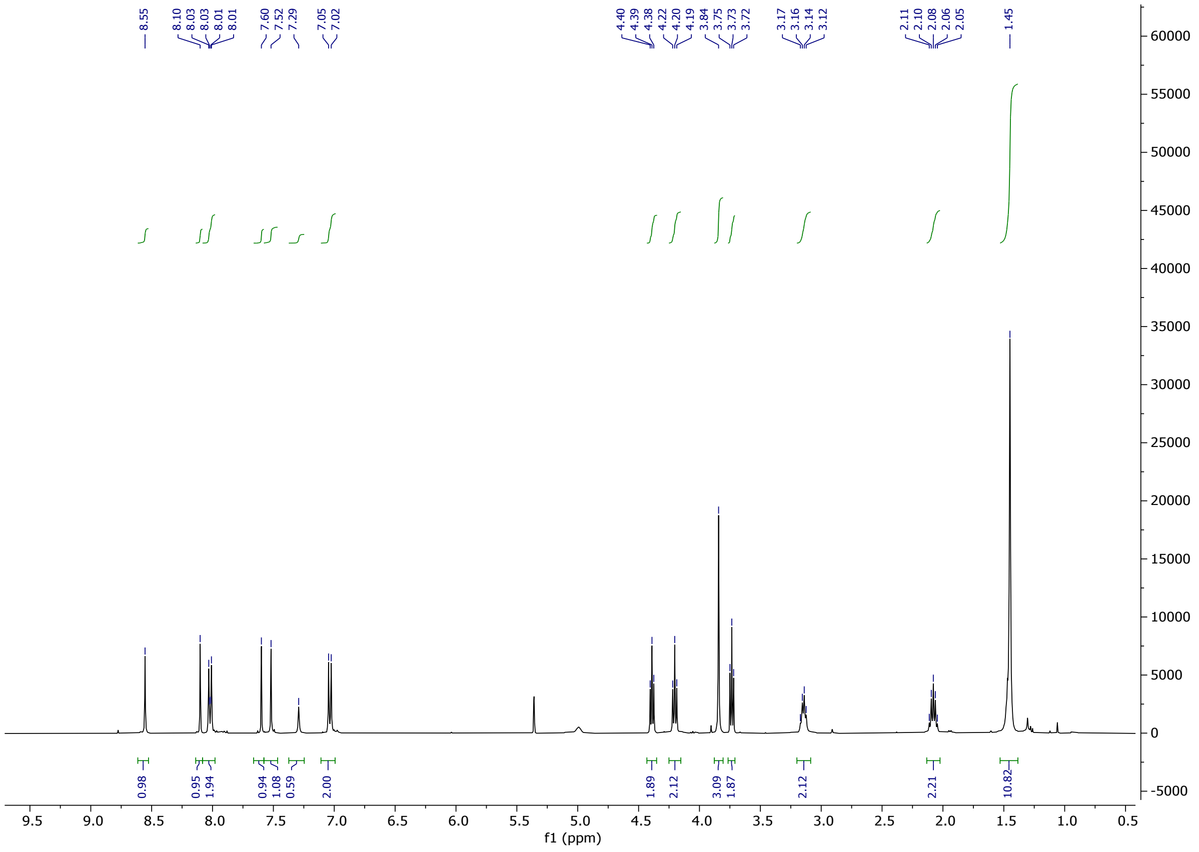

^{1}H spectrum (CD_2Cl_2, 400 MHz, 298 K) of **intermediate 16.**

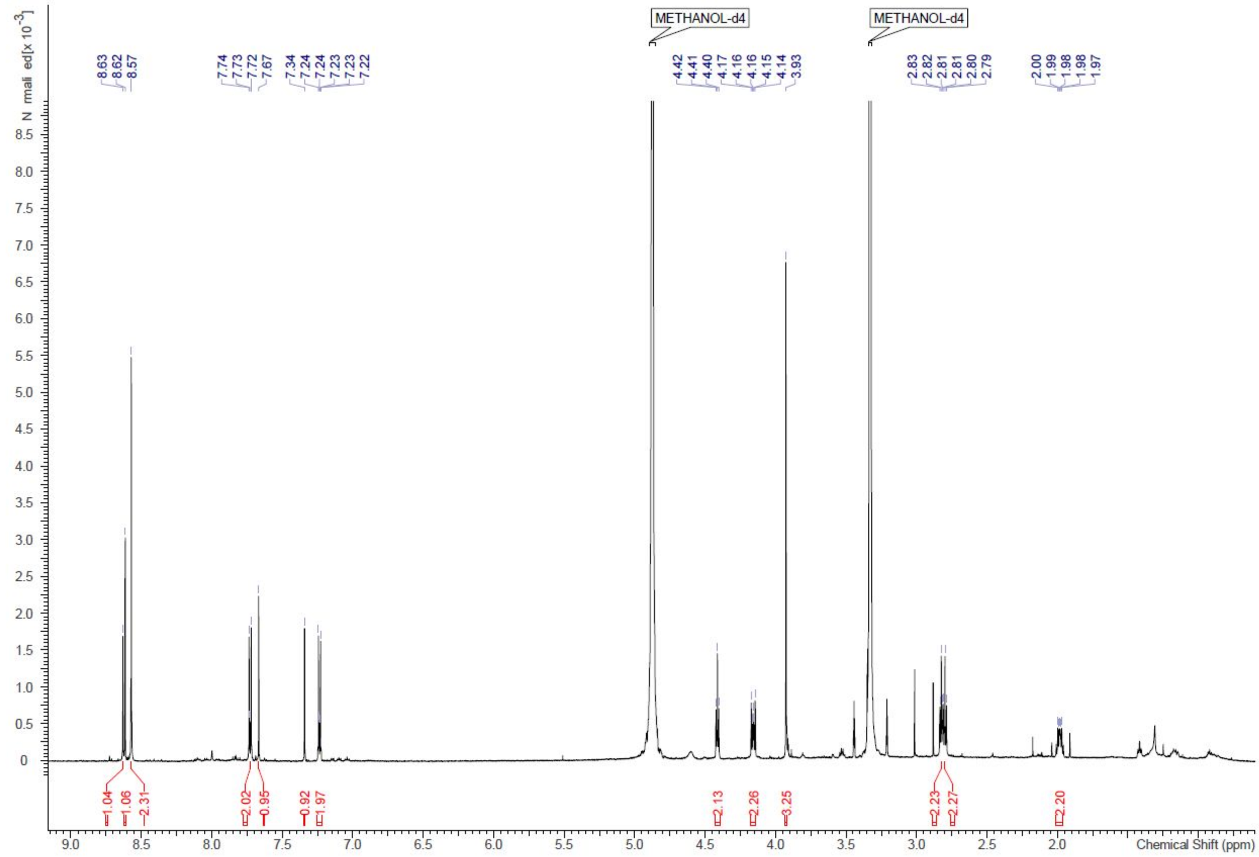

^1H spectrum (CD_3OD, 400 MHz, 298 K) of **macrocycle 5a.**

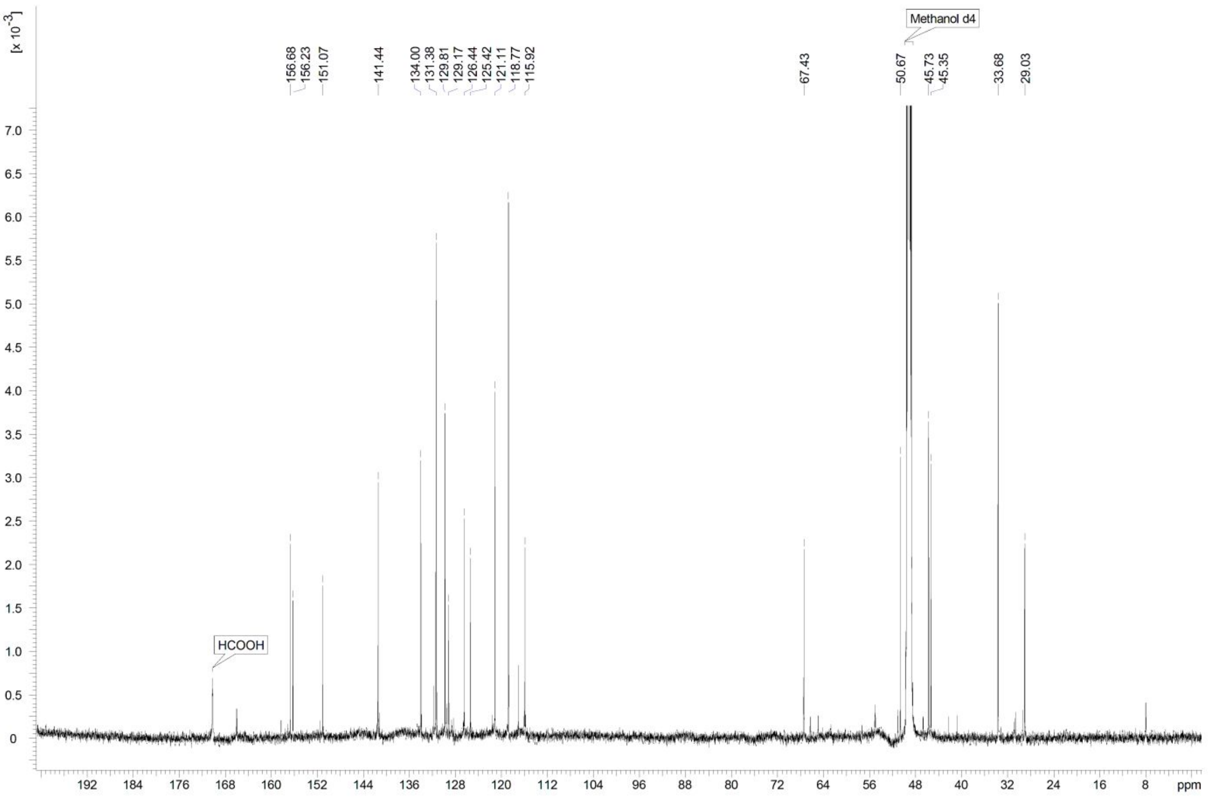

^{13}C spectrum (CD_3OD, 400 MHz, 298 K) of **macrocycle 5a**

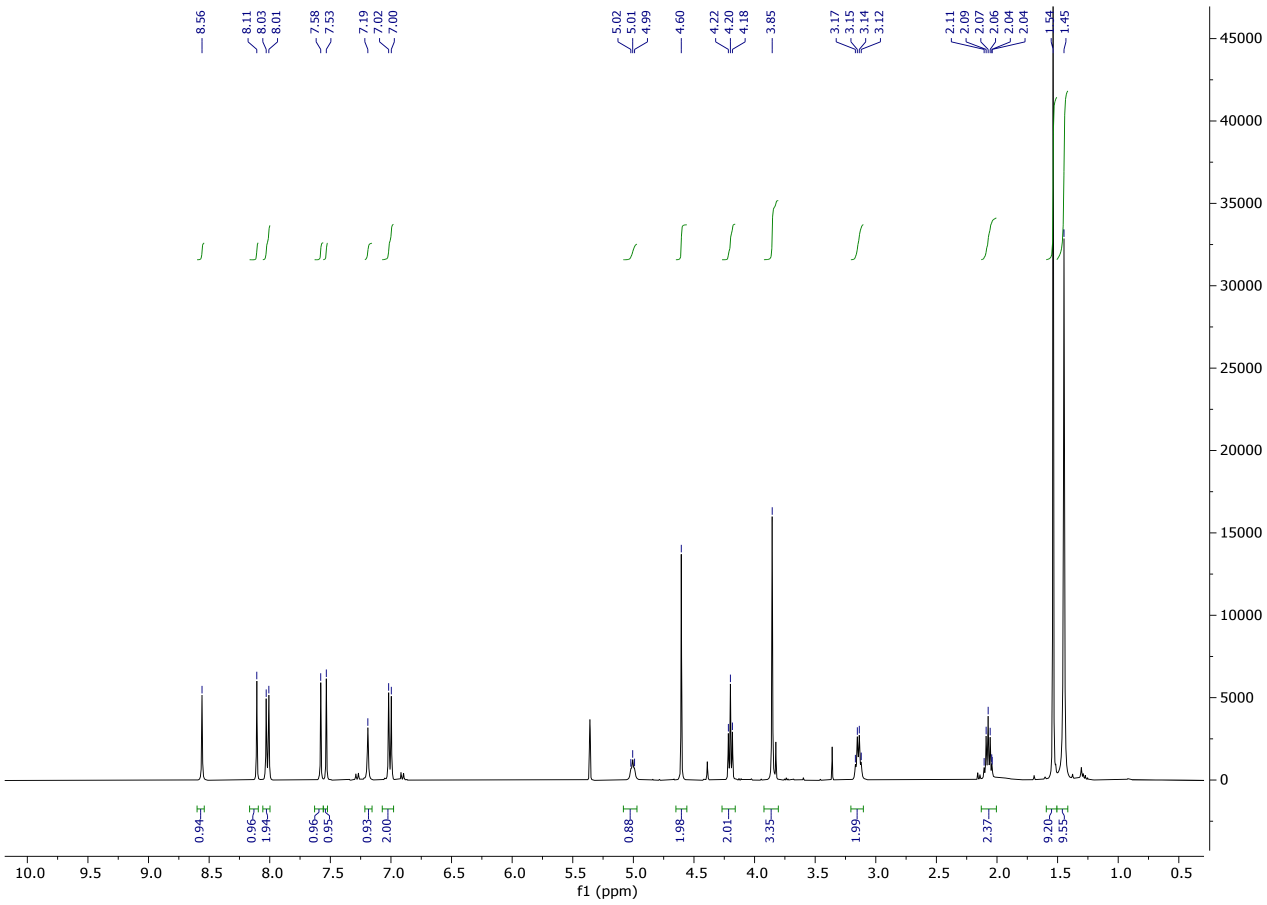

^{1}H spectrum (CD_2Cl_2, 400 MHz, 298 K) of **intermediate 18**.

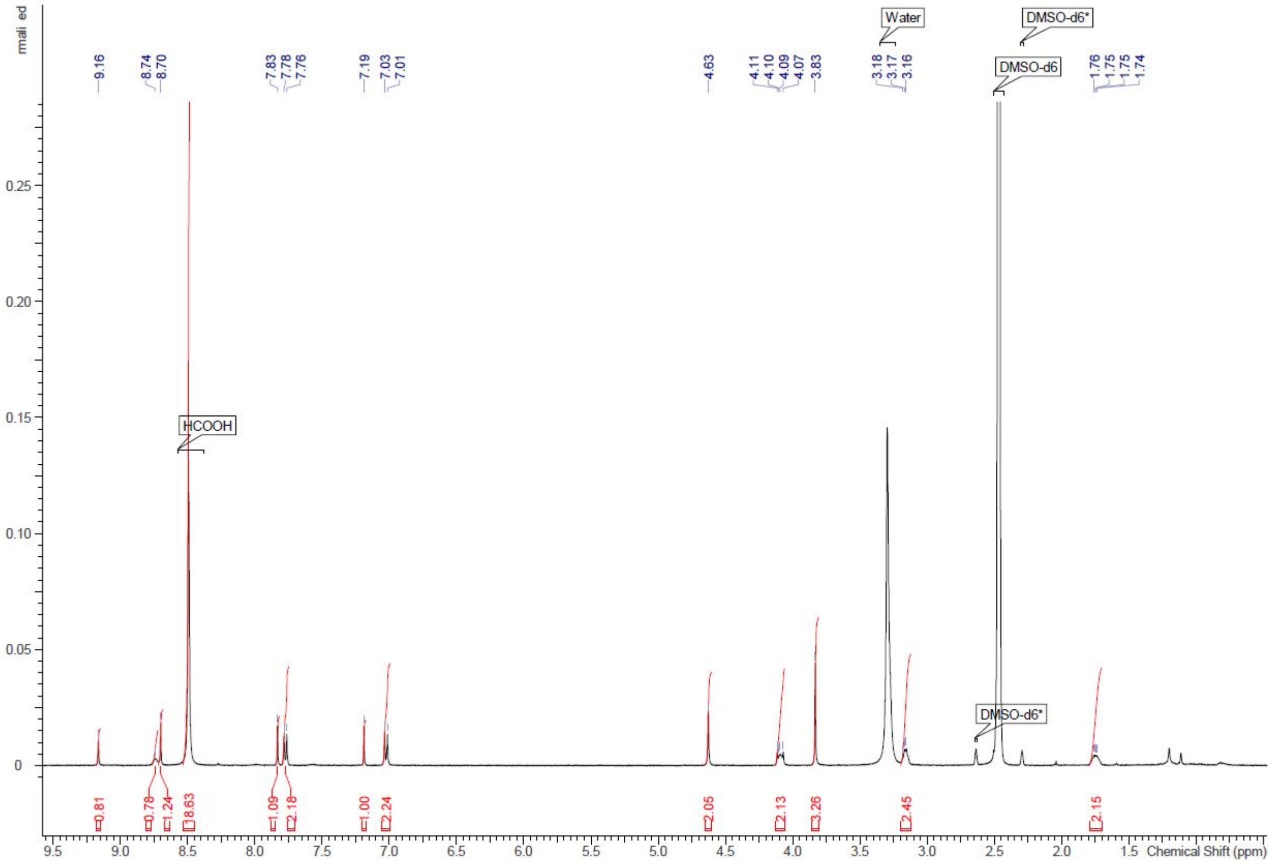

^{1}H spectrum (DMSO-d6, 400 MHz, 298 K) of **macrocycle 5b**.

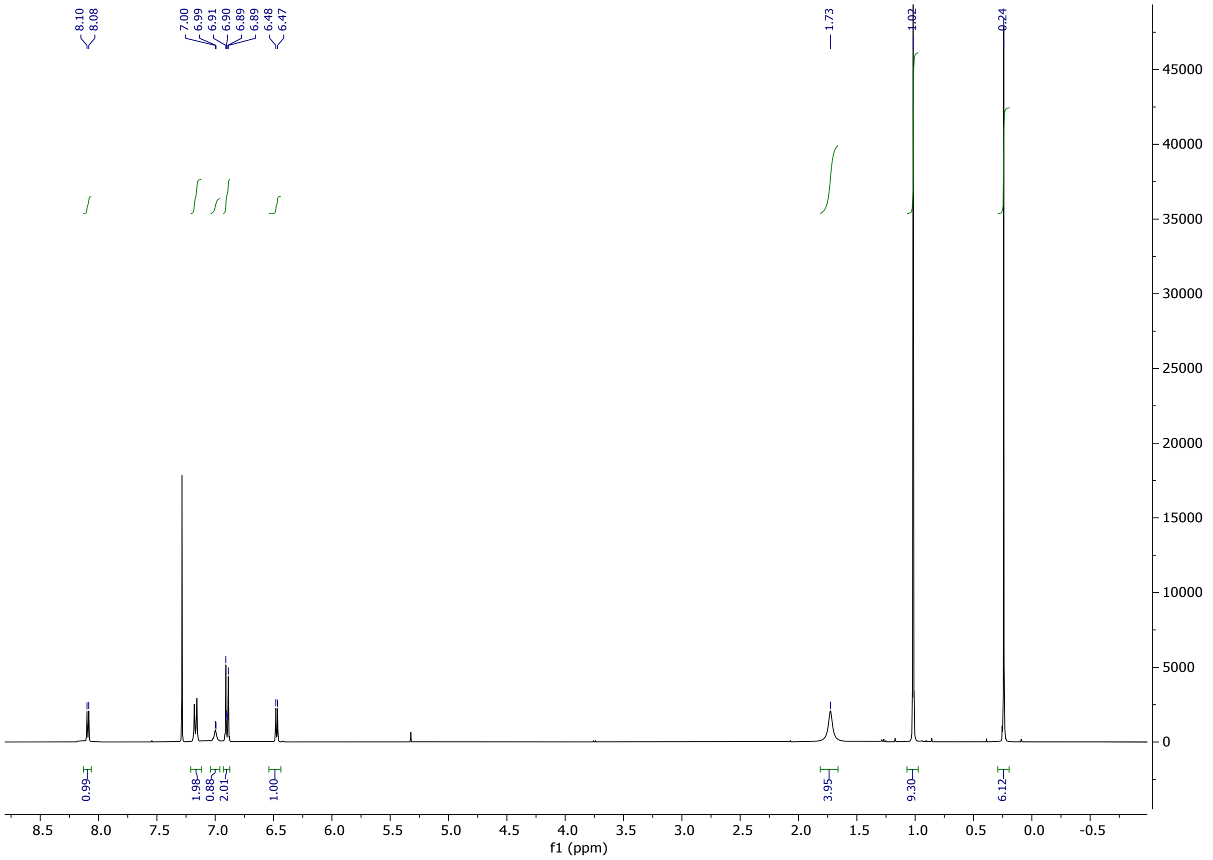

^{1}H spectrum ($CDCl_3$, 400 MHz, 298 K) of **intermediate 22.**

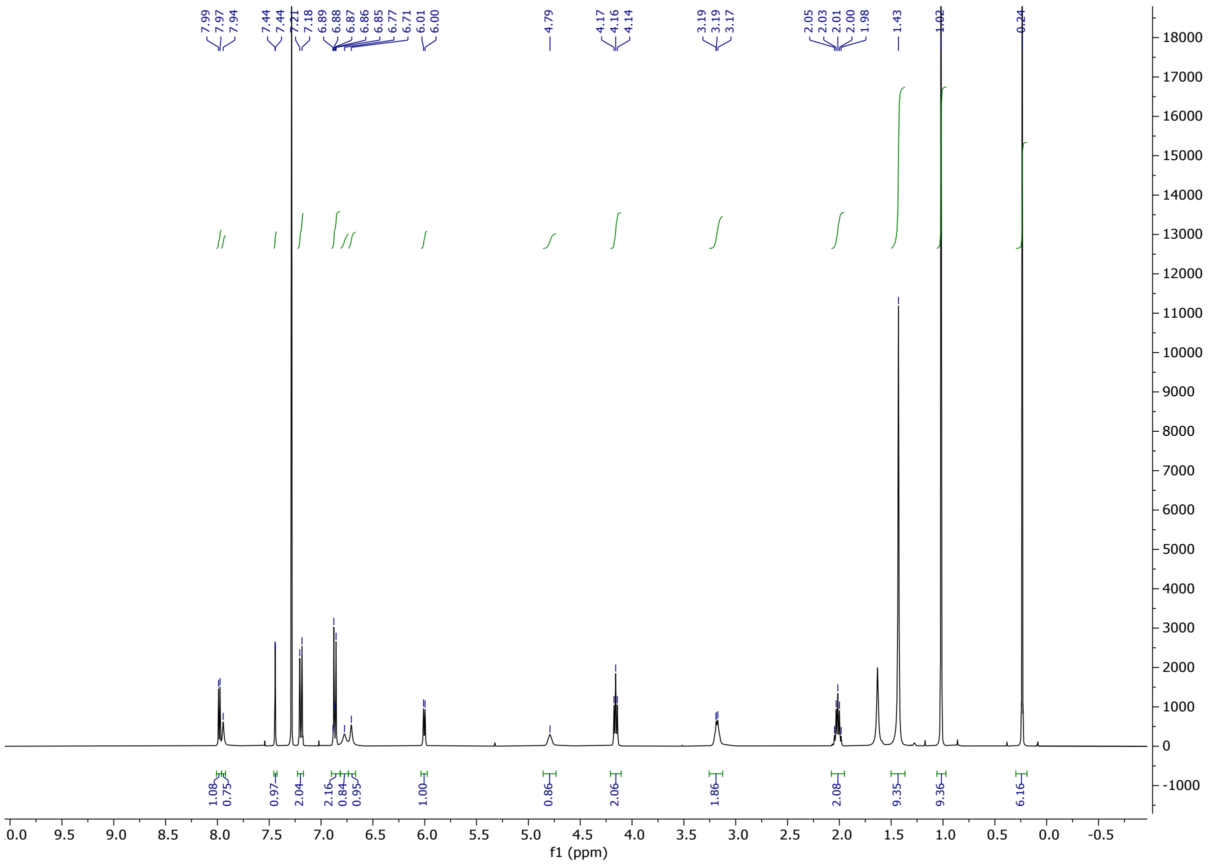

^{1}H spectrum ($CDCl_3$, 400 MHz, 298 K) of **intermediate 23.**

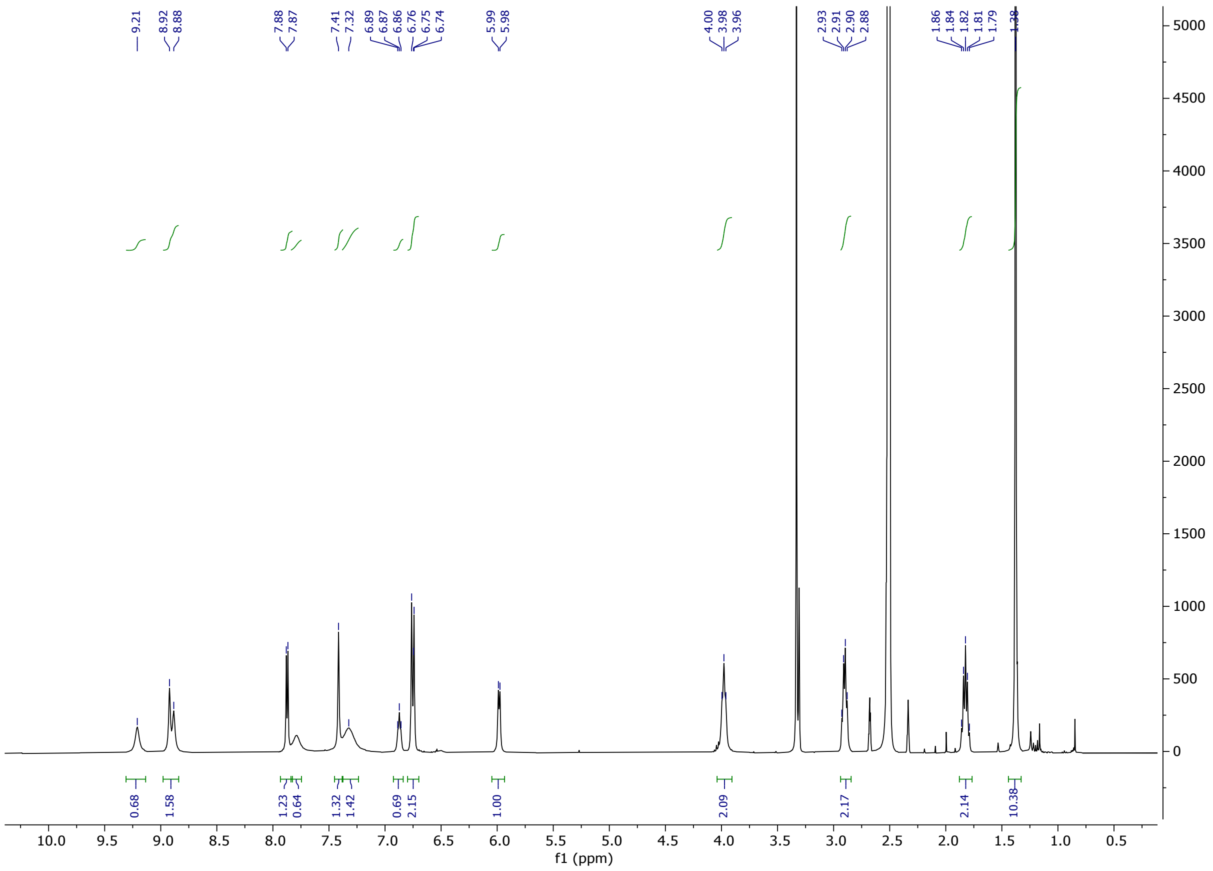

^{1}H spectrum (DMSO-d6, 400 MHz, 298 K) of **intermediate 24.**

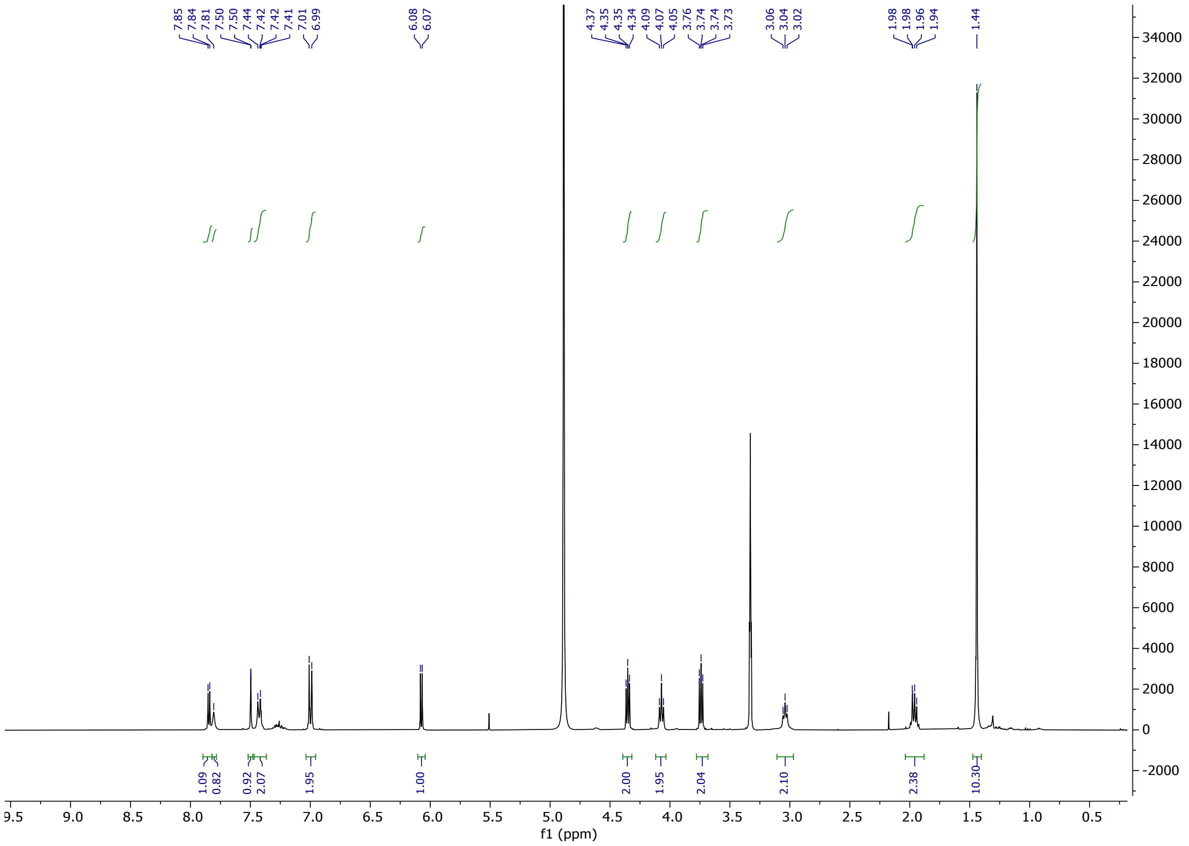

^{1}H spectrum (CD_3OD, 400 MHz, 298 K) of **intermediate 25**.

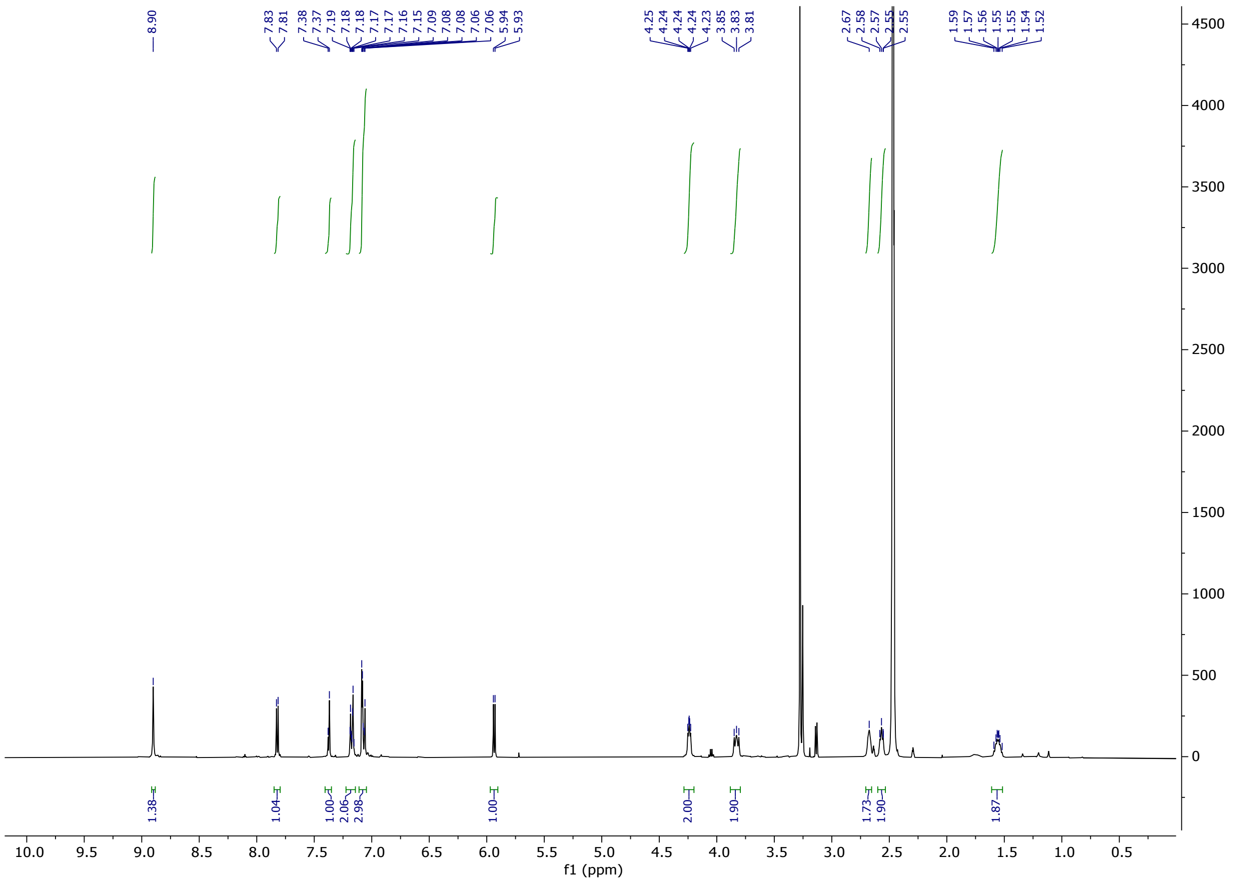

^{1}H spectrum (DMSO-d6, 400 MHz, 298 K) of **macrocycle 6a.**

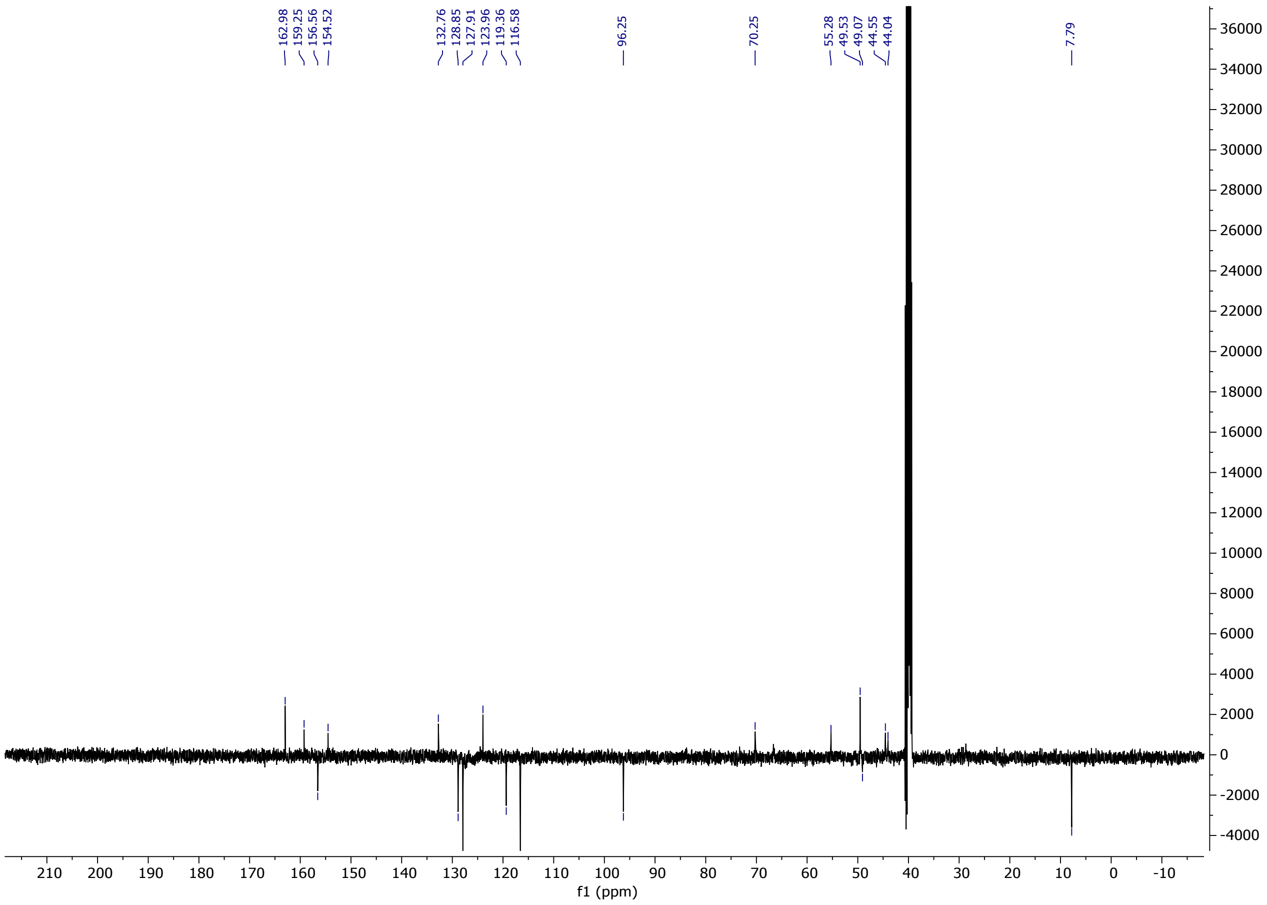

DEPTQ spectrum (DMSO-d6, 400 MHz, 298 K) of **macrocycle 6a.** Positive peaks are CH_2 - quaternary C, negative peaks are CH_3 - CH.

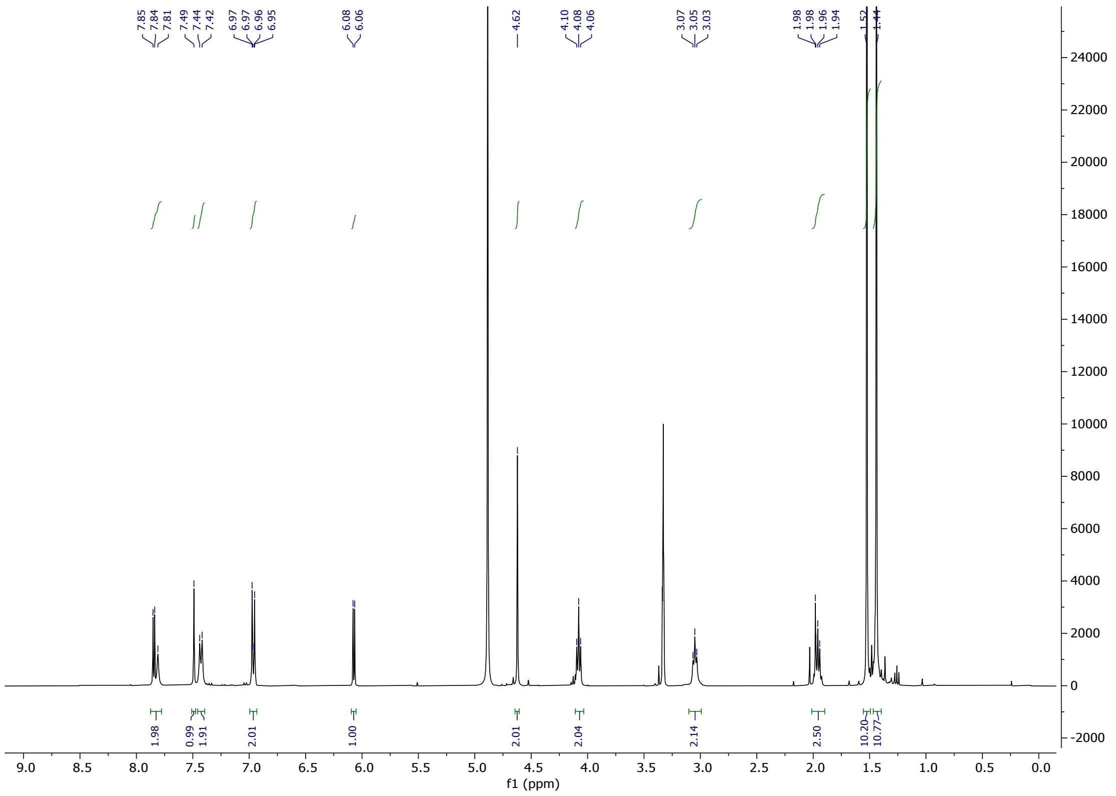

^{1}H spectrum (CD_3OD, 400 MHz, 298 K) of **intermediate 27.**

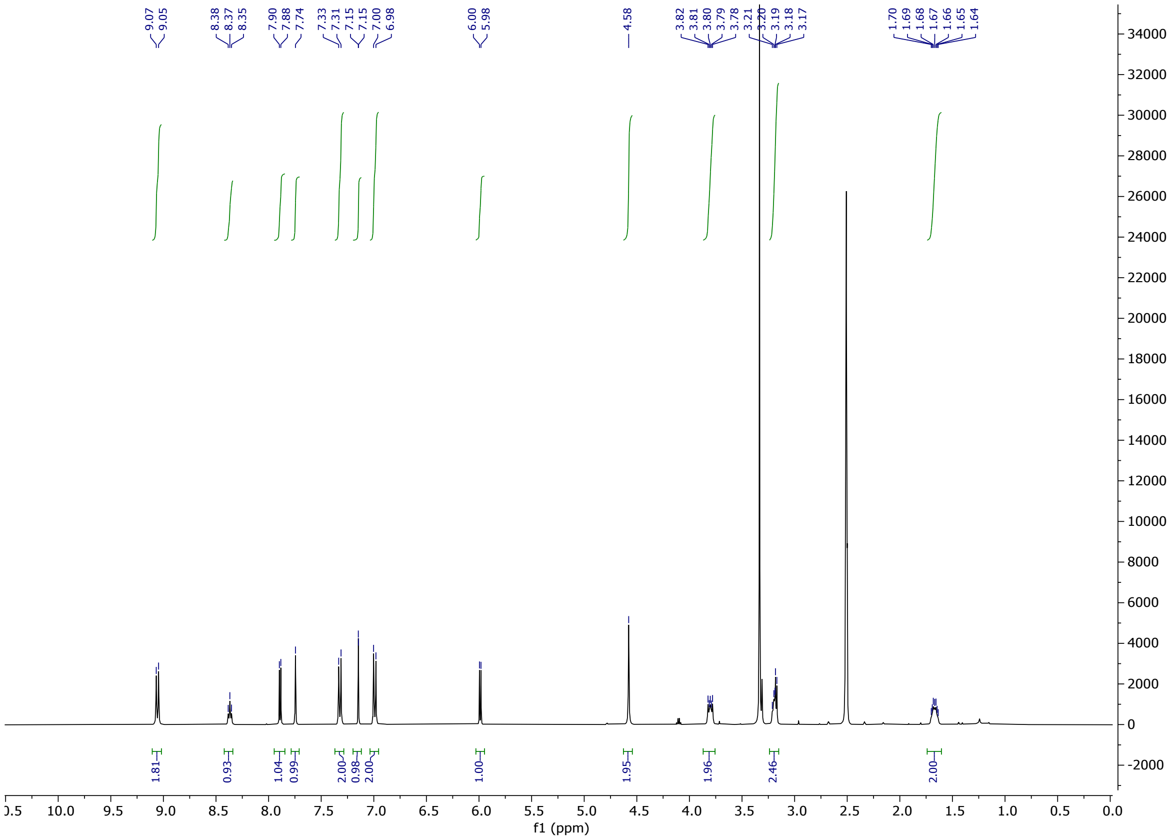

^{1}H spectrum (DMSO-d6, 400 MHz, 298 K) of **macrocycle 6b**

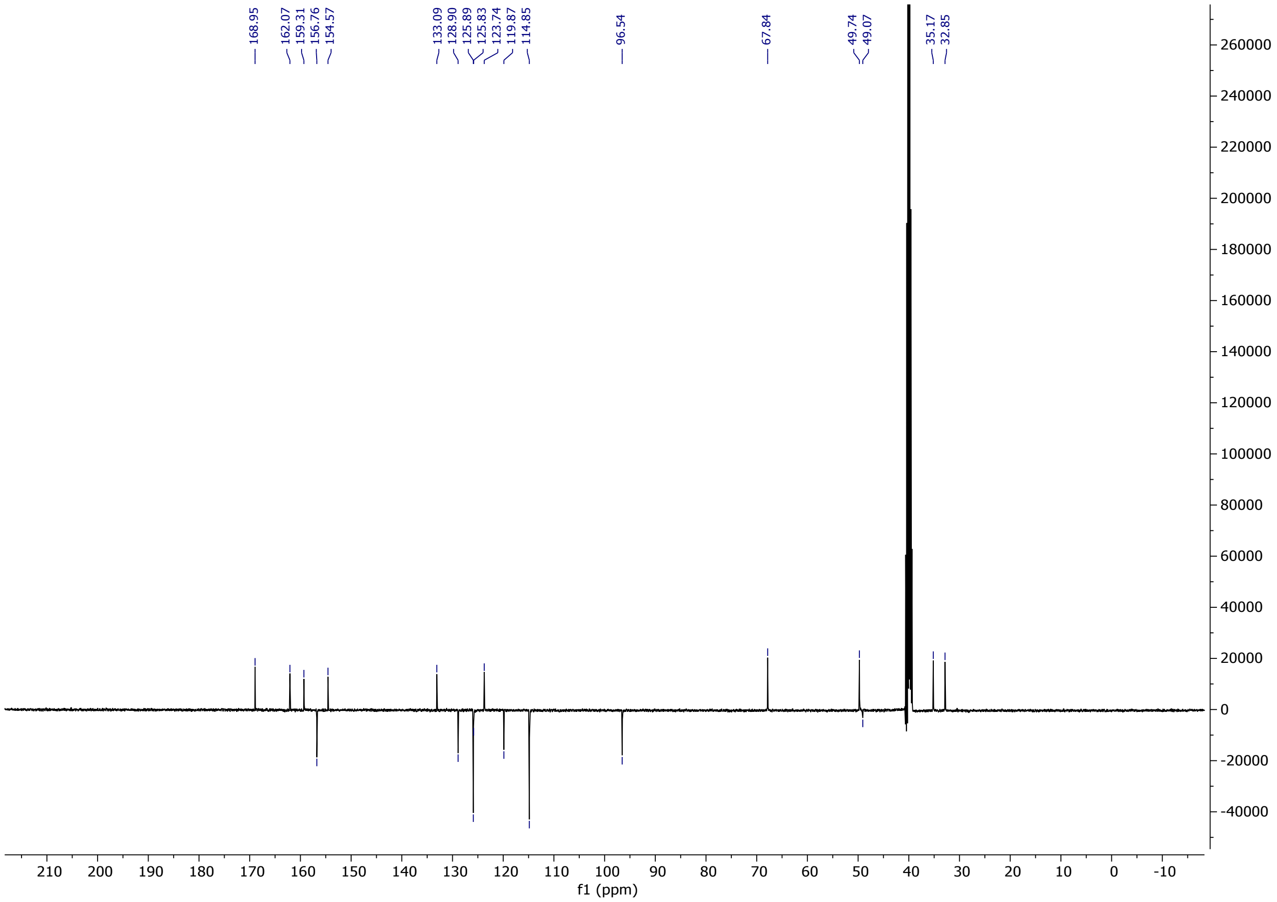

DEPTQ spectrum (DMSO-d6, 400 MHz, 298 K) of **macrocycle 6b.** Positive peaks are CH_2 - quaternary C, negative peaks are CH_3 - CH.

References

(a) Gilmer, J.; Schoenholz, S. S.; Riley, P. F.; Vinyals, O.; Dahl, G. E. Neural Message Passing for Quantum Chemistry. In *34th International Conference on Machine Learning, Proceedings of ICML 2017 - Volume 70*, Sydney, Australia, August 6-11, 2017.

(b) Vinyals, O.; Bengio, S.; Kudlur, M. Order Matters: Sequence to Sequence for Sets. In *4th International Conference on Learning Representations*, *Proceedings of ICLR 2016,* San Juan, Puerto Rico, May 2-4, 2016.

(c) Kingma, D. P.; Ba, J. Adam: A Method for Stochastic Optimization. In *3th International Conference on Learning Representations*, *Proceedings of ICLR 2015*, San Diego, CA, USA, May 7-9, 2015.

(d) Ermondi, G.; Jimenez, D. G.; Rossi Sebastiano, M.; Kihlberg, J.; Caron, G. Conformational Sampling Deciphers the Chameleonic Properties of a VHL-Based Degrader. *Pharmaceutics* **2023**, *15* (1), 272. https://doi.org/10.3390/pharmaceutics15010272.

(e) Ermondi, G.; Vallaro, M.; Goetz, G.; Shalaeva, M.; Caron, G. Updating the Portfolio of Physicochemical Descriptors Related to Permeability in the beyond the Rule of 5 Chemical Space. *Eur. J. Pharm. Sci.* **2020**, *146* , 105274. DOI: 10.1016/j.ejps.2020.105274.

(f) Goetz, G. H.; Philippe, L.; Shapiro, M. J. EPSA: A Novel Supercritical Fluid Chromatography Technique Enabling the Design of Permeable Cyclic Peptides. *ACS Med. Chem. Lett.* **2014**, *5* (10), 1167–1172. DOI: 10.1021/ml500239m.

(g) Garcia Jimenez, D.; Vallaro, M.; Rossi Sebastiano, M.; Apprato, G.; D'Agostini, G.; Rossetti, P.; Ermondi, G.; Caron, G. Chamelogk: A Chromatographic Chameleonicity Quantifier to Design Orally Bioavailable Beyond-Rule-of-5 Drugs. *J. Med. Chem.* **2023**, *66* (15), 10681–10693. DOI: 10.1021/acs.jmedchem.3c00823.
